# Nrf2 alleviates spaceflight-induced immunosuppression and thrombotic microangiopathy in mice

**DOI:** 10.1038/s42003-023-05251-w

**Published:** 2023-08-25

**Authors:** Ritsuko Shimizu, Ikuo Hirano, Atsushi Hasegawa, Mikiko Suzuki, Akihito Otsuki, Keiko Taguchi, Fumiki Katsuoka, Akira Uruno, Norio Suzuki, Akane Yumoto, Risa Okada, Masaki Shirakawa, Dai Shiba, Satoru Takahashi, Takafumi Suzuki, Masayuki Yamamoto

**Affiliations:** 1 Tohoku Medical Megabank Organization, Tohoku University, Sendai, Japan; 2 Department of Molecular Hematology, Tohoku University Graduate School of Medicine, Sendai, Japan; 3 The Advanced Research Center for Innovations in Next-Generation Medicine (INGEM) Tohoku University, Sendai, Japan; 4 Division of Oxygen Biology, New Industry Creation hatchery Center (NICHe), Tohoku University, Sendai, Japan; 5 Japanese Experiment Module (JEM) Utilization Center, Human Spaceflight Technology Directorate, Japan Aerospace Exploration Agency (JAXA), Tsukuba, Japan; 6 Department of Anatomy and Embryology and Laboratory Animal Resource Center in Transborder Medical Research Center, Institute of Medicine, University of Tsukuba, Tsukuba, Japan

**Keywords:** Coagulation system, Chronic inflammation

## Abstract

Spaceflight-related stresses impact health via various body systems, including the haematopoietic and immune systems, with effects ranging from moderate alterations of homoeostasis to serious illness. Oxidative stress appears to be involved in these changes, and the transcription factor Nrf2, which regulates expression of a set of cytoprotective and antioxidative stress response genes, has been implicated in the response to spaceflight-induced stresses. Here, we show through analyses of mice from the MHU-3 project, in which Nrf2-knockout mice travelled in space for 31 days, that mice lacking Nrf2 suffer more seriously from spaceflight-induced immunosuppression than wild-type mice. We discovered that a one-month spaceflight-triggered the expression of tissue inflammatory marker genes in wild-type mice, an effect that was even more pronounced in the absence of Nrf2. Concomitant with induction of inflammatory conditions, the consumption of coagulation-fibrinolytic factors and platelets was elevated by spaceflight and further accelerated by Nrf2 deficiency. These results highlight that Nrf2 mitigates spaceflight-induced inflammation, subsequent immunosuppression, and thrombotic microangiopathy. These observations reveal a new strategy to relieve health problems encountered during spaceflight.

## Abstract (web summary)

A spaceflight study shows that Nrf2 contributes to the maintenance of the immune system during spaceflight and suggests that improving the level of baseline Nrf2 activity alleviates the degree of immune suppression during spaceflight.

## Introduction

Recent studies on spaceflight biology have led to progress in the understanding that under unusual environmental conditions, such as the extreme radiation and microgravity conditions in space, animals suffer from a variety of pathophysiologic disturbances. To prepare for the next era of extended space travel, precise assessment of the impact of space travel on the human body becomes crucial. The radiation exposure dose during a stay at the International Space Station (ISS) is estimated to be 150 mSv/year^1^, which is 70 times higher than the average dose in Japan. Persistent low-dose irradiation causes the accumulation of reactive oxygen species (ROS)^2–4^. Considering the many lines of evidence for ROS-triggered cellular inflammation and subsequent immune dysfunction^5^, regulating ROS metabolism seems to be one of the most beneficial strategies to protect the body from persistent radiation injuries during space travel.

Microgravity causes alterations in fluid distribution in the body. While incremental changes in arterial pressure are well controlled when the body is in an upright position under normal gravity, this gradient becomes uniform throughout the body in space, which puts a burden on the central cardiovascular system^6,7^. This gives rise to a fluid shift, elicits significant elevation of the microvascular pressure in the central compartment and head, and causes symptoms in the central nervous system through intracranial hypertension^8^. Notably, previous studies revealed that the overall circulating plasma volume is reduced during spaceflights, resulting in increases in red blood cell (RBC) parameters as a result of haemoconcentration^9^, while RBC destruction is accelerated by spaceflight stress^10^. The polycythemic state is gradually reversed through haematopoietic adaptation to microgravity during a stay in space^11^. In contrast, as a result of fluid redistribution during adaptation to ground gravity, astronauts returning from space develop genuine anaemia^12^.

It has been shown that in addition to cosmic radiation, alterations in gravity also generate ROS in cells^13^, which may lead to the initiation and progression of inflammation^5^. Taken together, these observations from space biology studies support the notion that microgravity-induced mechanical stress and radiation-induced oxidative stress converge to affect the body, and the combined impact of these two stresses may lead to sequelae after the space mission, such as a higher incident rate of cardiovascular disease^14^, neuro-ocular disease^15^ and/or persistent haemolysis^10^.

The transcription factor Nrf2 (NF-E2-related factor-2) is a master regulator of defence pathways that respond to various stresses, including oxidative, mechanical and toxic chemical (often electrophilic) stresses^16^. Upon an increase in cellular ROS levels, Keap1 (Kelch-like ECH-associated protein 1) senses the increase in ROS levels and stops the ubiquitination of Nrf2; therefore, Nrf2 escapes proteasomal proteolysis and rapidly accumulates in the nucleus. Nrf2 upregulates a set of cytoprotective genes encoding detoxification enzymes and antioxidant enzymes that scavenge ROS^16^.

We hypothesised that Nrf2 may protect against space-related stresses. To address this hypothesis, we designed and conducted the Mouse Habitat Unit-3 [MHU-3] project, in which six Nrf2-knockout (KO)^17^ and six wild-type (WT) mice travelled to the ISS and stayed there for 31 days^18^. Initial analyses of the mice demonstrated that spaceflight indeed significantly increased the expression of Nrf2 target genes. The analyses further revealed that Nrf2 differentially affects spaceflight-triggered signalling pathways^18^. For instance, we found that Nrf2 preserves homoeostasis of skeletal muscle, kidney tissue and epididymal white adipose tissue^19–21^.

In this regard, while it has been shown that space travel influences red cell indices and immune system^10,12,22^, the details of the underlying mechanisms remain to be clarified. Furthermore, the relationship between haematopoietic and immunological changes and Nrf2 function has not been addressed. In this study, therefore, we aimed to extend the analyses of the mice from the MHU-3 mission, with a special focus on haematological and immunological assessments, along with a number of experimental challenges to overcome difficulties inherent to the space study. Interestingly, the haemoconcentration caused by microgravity in astronauts was not observed in quadruped mice during spaceflight, while an increase in RBC mass and repression of erythroid-gene expression were evident after returning to the ground, irrespective of Nrf2 expression. Spaceflight markedly changed platelet physiology, concomitant with an increase in platelet turnover status. In stark contrast, the expression of immune-related genes was repressed by spaceflight in an Nrf2-dependent manner. This study unequivocally demonstrates that Nrf2 alleviates spaceflight-mediated inflammation and subsequent immune repression and thrombotic microangiopathy.

## Results

### Haematocrit values increased in mice after spaceflight

**Fig. 1:**
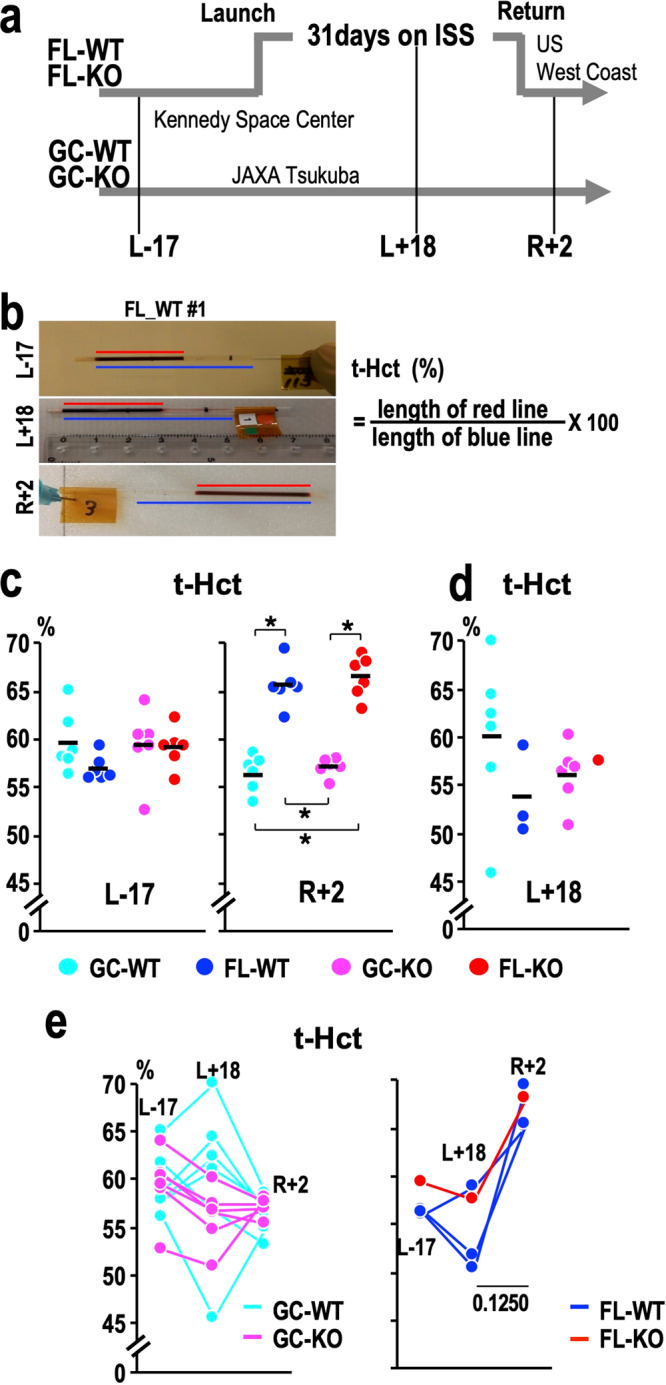
Haematocrit of tail vein blood. **a** Overview of the MHU-3 project, in which tail vein blood samples were collected from mice. **b** Representative pictures of the centrifuge tubes used for measuring tail-HCT values (t-Hct). Red and blue lines indicate the lengths of packed RBCs and whole blood, respectively. **c** Dot plots of t-Hct at L-17 (left) and R + 2 (right). *p < 0.05. **d** Dot plots of t-Hct at L + 18. **e** Time course of the changes in t-Hct at L-17, L + 18 and R + 2. Data from 6 GC-WT and 6 GC-KO mice are shown in the left panel, and those from 3 FL-WT and 1 FL-KO mice are shown in the right panel. The *P*-value obtained with the two-tailed Wilcoxon signed-rank test for the comparison between the values obtained at L + 18 and R + 2 in FL mice is shown. Dots represent individual animals in (**c**) (**d**) and (**e**). Means are shown in the plots in (**d**) and (**e**).

In the MHU-3 mission, we sent 6 male wild-type and 6 male Nrf2-KO mice to space (FL-WT and FL-KO mice, respectively) (Fig. 1a)^18^. These mice stayed at the ISS for 31 days. As a ground control, we used the same number of male wild-type and Nrf2-KO mice (GC-WT and GC-KO mice, respectively). The ground control experiment was conducted with utmost precision to simulate the conditions of the flight experiment, which was achieved by employing identical single cage housing technology, laboratory food and water supply, as well as the same system to monitor mouse food consumption and water intake^18,21^. Tail blood samples from the FL mice were collected into capillary tubes 17 days before launch (L-17), 18 days after launch while in space (L + 18), and 2 days after landing (R + 2). Tail blood samples from GC mice were also collected on the corresponding days. Blood samples could not be collected from one FL-WT mouse at L + 18 due to a minor tail injury and the veterinarian’s decision^21^. After collecting the blood, the capillary tubes were immediately centrifuged in the ISS. We took pictures of the centrifuge tubes (Fig. 1b) to measure the lengths of whole blood and packed red blood cells (RBCs), as shown by red and blue lines, respectively (Fig. 1b, Supplementary Fig. 1). We referred to the percent length of packed RBCs (red line vs. blue line) as the tail haematocrit value (t-Hct) (Fig. 1b).

Of note, the t-Hct of the FL mice at R + 2 was significantly increased compared with that of the GC mice at R + 2 (Fig. 1c). This change was also observed in Nrf2-KO mice, but the t-Hct before launch (L-17) did not change substantially in all four groups of mice (Fig. 1c). As we obtained tail blood from the same set of mice during their stay at the ISS (L + 18), we attempted to examine the HCT at L + 18. Regrettably, however, the t-Hct of seven FL mice could not be determined since the edges of the whole blood were inadvertently hidden by labels (Supplementary Fig. 1). Nonetheless, we could measure the t-Hct of three FL-WT and one FL-KO mice. As shown in Fig. 1d, the t-Hct of these mice at L + 18 was comparable to that of the GC-WT and GC-KO mice. The time-course alterations of the four FL mice demonstrated a trend of increasing t-Hct after landing but no substantial changes in t-Hct between L-17 and L + 18 (Fig. 1e). These findings demonstrate that the transition from normal gravity to microgravity and continued exposure to microgravity do not alter t-Hct, but the transition from microgravity to normal gravity gives rise to an increase in t-Hct.

### Increases in RBC number and RDW in mice after spaceflight

To verify the changes in the haematopoietic indices of mice after spaceflight, we collected blood from the inferior vena cava (IVC) of anaesthetised mice using EDTA-coated syringes immediately before euthanasia after the return to ground at R + 2. The blood samples were kept on ice, and the haematopoietic indices of the FL mice were measured with a haemocytometer in a laboratory located on the US West Coast within 6.5 h after blood collection, while those of the GC mice were measured in Japan.

**Fig. 2:**
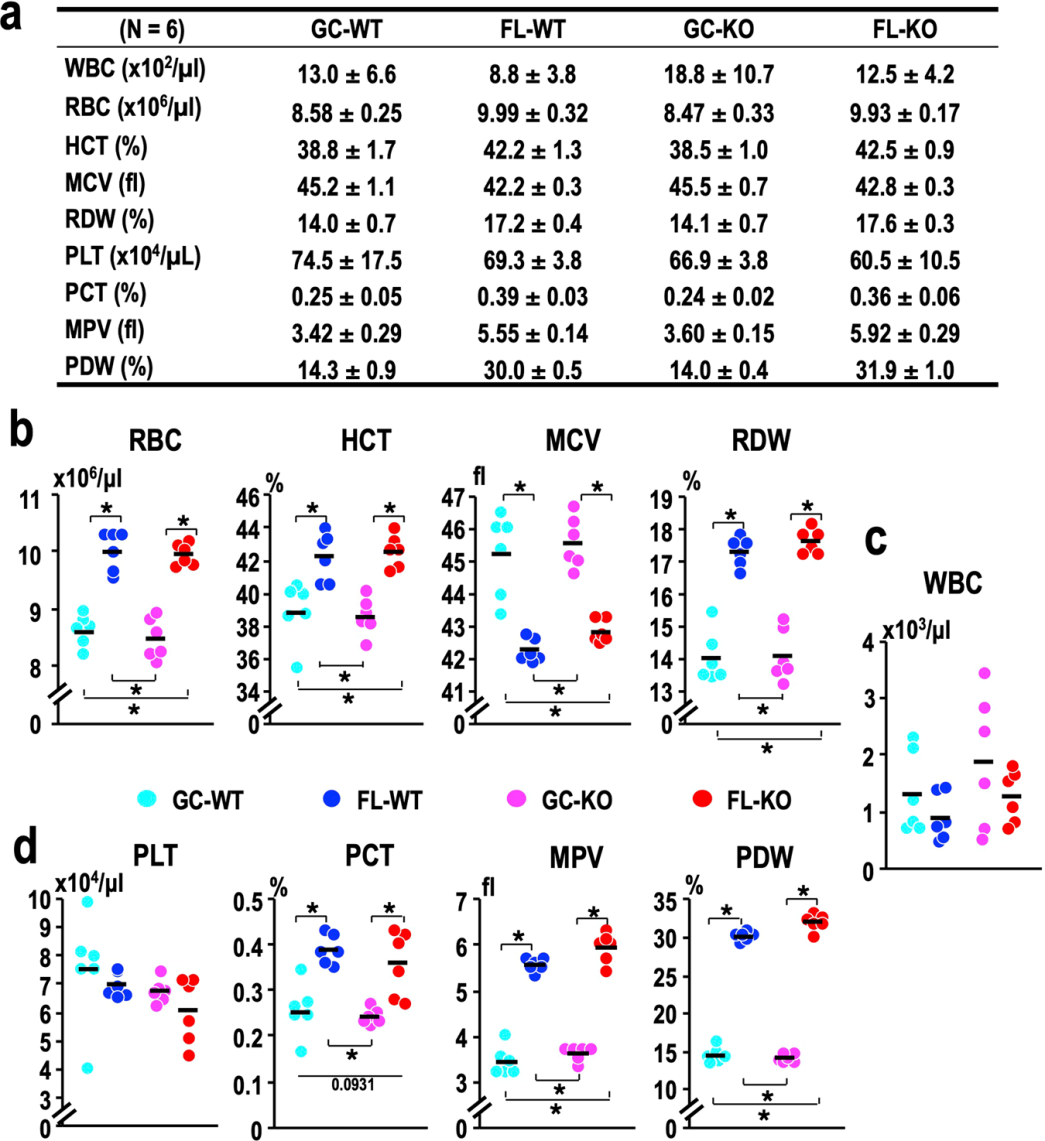
Evaluation of inferior vena cava blood parameters. **a** Summary of haematopoietic indices. Mean values and standard deviations are shown. **b–d** Dot plots of the indicated values for erythroid parameters (**b**), white blood cell counts (**c**), and platelet parameters (**d**). Dots represent individual animals. Means are shown in the plots. *p < 0.05.

The haematopoietic indices are shown in Fig. 2a. Consistent with the t-Hct results, the haematocrit (Hct) values of the IVC samples were significantly elevated concomitant with the RBC count in the FL mouse groups, regardless of the genotype (Fig. 2b). Importantly, the values of red blood distribution width (RDW), which is an index of the heterogeneity of RBC size, were significantly elevated in FL-WT and FL-KO mice, accompanied by a decrease in mean corpuscular volume (MCV) (Fig. 2b). These results suggest that the RBCs of FL mice exhibit the features of microcytosis and anisocytosis. The increase in RDW is known to be a prognostic marker for various diseases, such as oncologic diseases^23^, chronic kidney diseases^24^, cardiovascular diseases^25^, and severe infection^26,27^. In contrast, we did not observe substantial changes in the white blood cell (WBC) count (Fig. 2c). These results thus demonstrate that spaceflight causes abnormalities in RBC size and number in mice.

Unfortunately, the haemocytometer in the US laboratory could not properly evaluate the haemoglobin concentration due to an unknown problem(s). Therefore, we could not determine the other two Wintrobe’s indices.

### Increase in platelet size in mice after spaceflight

We next examined platelet indices. The number of platelets did not change substantially in response to spaceflight or Nrf2 gene knockout (Fig. 2d, left panel). This observation is in contrast to the previous observation, in which the platelet count was increased after landing^28^. The reason for this difference is not clear at present, but we surmise that the following points may be pertinent. While both studies were conducted utilising the C57BL/6 line of mice, the latter study carried out 12 days of spaceflight utilising female mice^28^, whereas our study carried out 31 days of spaceflight utilising male mice.

We also found that the plateletcrit (PCT), which represents the volume occupied by platelets within the blood, was conspicuously elevated by spaceflight, and consequently, the mean platelet volume (MPV) was significantly elevated in FL mice, regardless of the presence or absence of Nrf2 (Fig. 2d). Furthermore, the platelet distribution width (PDW), an index of the heterogeneity of platelet size, was elevated in the FL samples from both WT and Nrf2-KO mice compared with the corresponding GC samples (Fig. 2d).

Throughout this study, we applied the Kruskal‒Wallis test to evaluate differences among the GC-WT, FL-WT, GC-KO and FL-KO groups, and subsequently employed Steel-Dwass multiple comparisons to evaluate differences of multiple pairs of groups. Given the considerable impact of spaceflight on the parameters tested, we aimed to integrate evaluations of the effects of Nrf2 status by Wilcoxon rank sum test, comparing the GC-WT vs. GC-KO and FL-WT vs. FL-KO, individually.

To evaluate the impact of Nrf2 states on platelet parameters in both ground and spaceflight conditions, we contrasted platelet parameter values between WT and Nrf2-KO mice under GC and FL conditions independently (Supplementary Fig. 3). Our alternative analysis produced consistent results, indicating that alterations in MPV and PVD values were specifically amplified by the Nrf2-KO status under spaceflight conditions. Taken together, these results confirm that spaceflight significantly impacts erythrocyte and platelet homoeostasis.

### Elevated expression of genes involved in coagulation and fibrinolysis in FL mice

The significant elevations in MPV and PDW suggested the presence of increased platelet turnover in FL mice^29^. In this regard, it should be noted that spaceflight changes lipid metabolism in mice, with elevated glycerophospholipid and sphingolipid levels in the plasma during spaceflight^21^. High plasma lipid concentrations have been suggested to correlate with a risk of venous thrombosis^30^. We further found in the integrated Biobank for Space Life Science (ibSLS) database (https://ibsls.megabank.tohoku.ac.jp/)^31^ that trimethylamine N-oxide (TMAO), a phosphatidylcholine-containing food-derived metabolite, is increased in the plasma during spaceflight (Supplementary Fig. 2). TMAO is associated with a risk of thrombotic events in humans via the induction of endothelial inflammatory injury and platelet activation^32,33^. Taking these wide-ranging observations into consideration, we hypothesised that spaceflight stress may activate thrombosis.

**Fig. 3:**
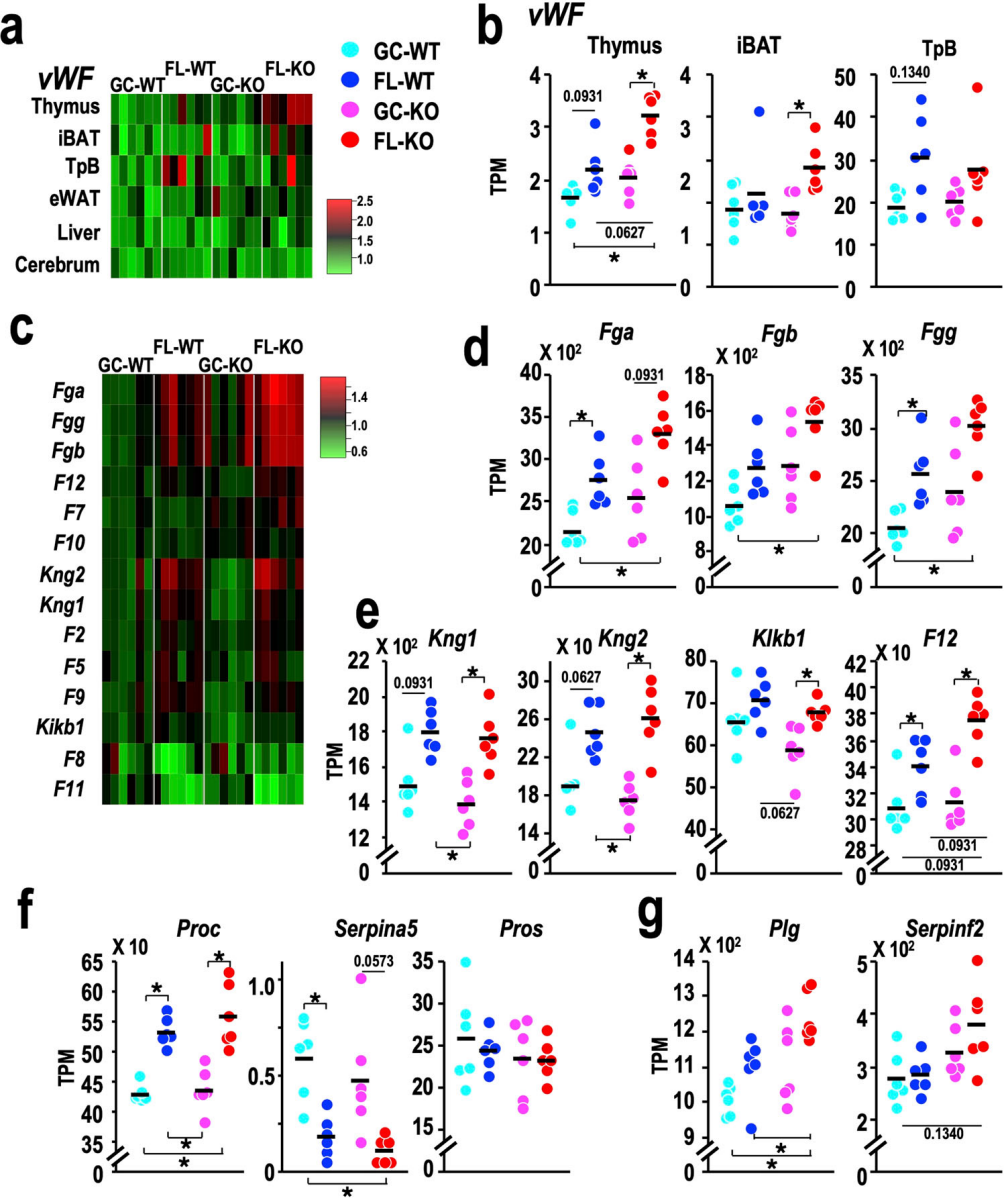
Gene expression changes related to coagulation and fibrinolysis. **a** Heatmap of the relative expression of the *vWF* gene in the thymus, interscapular brown adipose tissue (iBAT), temporal bone (TpB), epididymal white adipose tissue (eWAT), liver, and cerebrum of GC-WT, FL-WT, GC-KO and FL-KO mice. The mean value obtained for each gene in GC-WT mice was set to one. **b** Dot plot of *vWF* gene expression in the thymus, iBAT and TpB. **c** Heatmap of the relative expression of coagulation factor genes in the livers of GC-WT, FL-WT, GC-KO and FL-KO mice. The mean value obtained for each gene in GC-WT mice was set to one. **d** Dot plot of the expression of fibrinogen genes in the liver. **e** Dot plot of the expression of the *Kng1*, *Kng2*, *Klkb1* and *F12* genes in the liver. **f** Dot plot of the expression of the *Proc*, *Serpina5* and *Pros* genes in the liver. **g** Dot plot of the expression of the *Plg* and *Serpinf2* genes in the liver. Dots represent individual animals. Means are shown in the plots. *p < 0.05.

To address this hypothesis, we first examined the expression of the *vWF* gene encoding von Willebrand factor (vWF) in nonhaematopoietic tissues, i.e., the thymus, interscapular brown adipose tissues (iBAT), and temporal (Tp) bone areas, including the inner ear tissue, eWAT, liver and cerebrum, of mice in the ibSLS database. vWF is mainly produced in endothelial cells and megakaryocytes, and plasma vWF is concurrently increased with inflammation and involved in the pathogenesis of intravascular diseases^34^. We found that the expression level of *vWF* tended to be increased in a few WT-FL mouse tissues compared to WT-GC tissues (Fig. 3a). Notably*, vWF* gene expression was significantly elevated in the thymus and iBAT of FL-KO mice compared with GC-KO mice (Fig. 3b). This result was consistent in an alternative statistical analysis that directly compared the effect of Nrf2-KO under GC and FL conditions (Supplementary Fig. 4).

We then examined the expression of genes encoding tissue factor (coagulation factor III; F3), intercellular adhesion molecule 1 (ICAM1) and vascular cell adhesion molecule 1 (VCAM1), all of which are involved in endothelial activation^35,36^. Similar to the results obtained for the *vWF* gene, the expression of *Icam*1 and *Vcam1* tended to be elevated in the thymus and iBAT of FL-KO mice compared with GC-KO mice, and this trend was also clear in Nrf2-KO mice (Supplementary Fig. 5).

As the liver plays a pivotal role in the clotting process by synthesising coagulation factors, anticoagulants and fibrinolysis proteins, we next analysed the expression of coagulation factors in the liver using the ibSLS database. The expression of a variety of coagulation factors was found to be elevated in both FL-WT and FL-KO mice, and the elevations were markedly prominent in the FL-KO mice (Fig. 3c).

Upon examination of individual genes, we found that the levels of fibrinogen genes (α, β and γ) were increased by the spaceflight and the elevation of these genes became especially prominent in response to the combination of spaceflight and Nrf2-KO (Fig. 3d). This result appeared to be reproducible in the alternative statistical analysis (Supplementary Fig. 6a), indicating that spaceflight-induced fibrinogen gene activation was actually enhanced on the Nrf2-KO background. Elevated expression of the *Kng1*/*Kng2*, *Klkb1* and *F12* genes, which encode molecular kininogens, prekallikrein and coagulation factor XII, respectively, was also noted in the FL mice compared to the GC mice and was especially prominent on the Nrf2-KO background (Fig. 3e). Importantly, the alternative statistical analysis revealed that the changes in *F12* gene expression were specifically intensified by the Nrf2-KO status under spaceflight conditions (Supplementary Fig. 6b). These findings prompted us to hypothesise that the turnover of coagulation factors, especially factors involved in the early phase of the intrinsic coagulation cascade, is markedly increased in FL mice and the increase is magnified in the Nrf2-KO condition. Furthermore, expression of the *Proc* gene encoding protein C was significantly elevated in the livers of FL mice, while expression of the S*erpina5* gene encoding a protein C inhibitor was downregulated in the FL mice compared to the GC mice, and the expression of *ProS* encoding protein S was not changed among the four groups (Fig. 3f).

Intriguingly, expression of the *Plg* (plasminogen) gene was activated by spaceflight, and this activation was enhanced on the Nrf2-KO background. The expression of *Serpinf2* encoding α2-antiplamin also appeared to be increased by the combination of spaceflight and Nrf2-KO (Fig. 3g, Supplementary Fig. 6c). Taken together, these data show that spaceflight induces perturbations in congealing-fibrinogenolytic pathway gene expression, and these perturbations occur *en bloc*, leading to the activation of thrombosis and fibrinolytic susceptibility in FL mice, which appears to be worsened in the absence of Nrf2 activity.

### Spleen shrinkage accompanied by a reduction in cell number in the white pulp

**Fig. 4:**
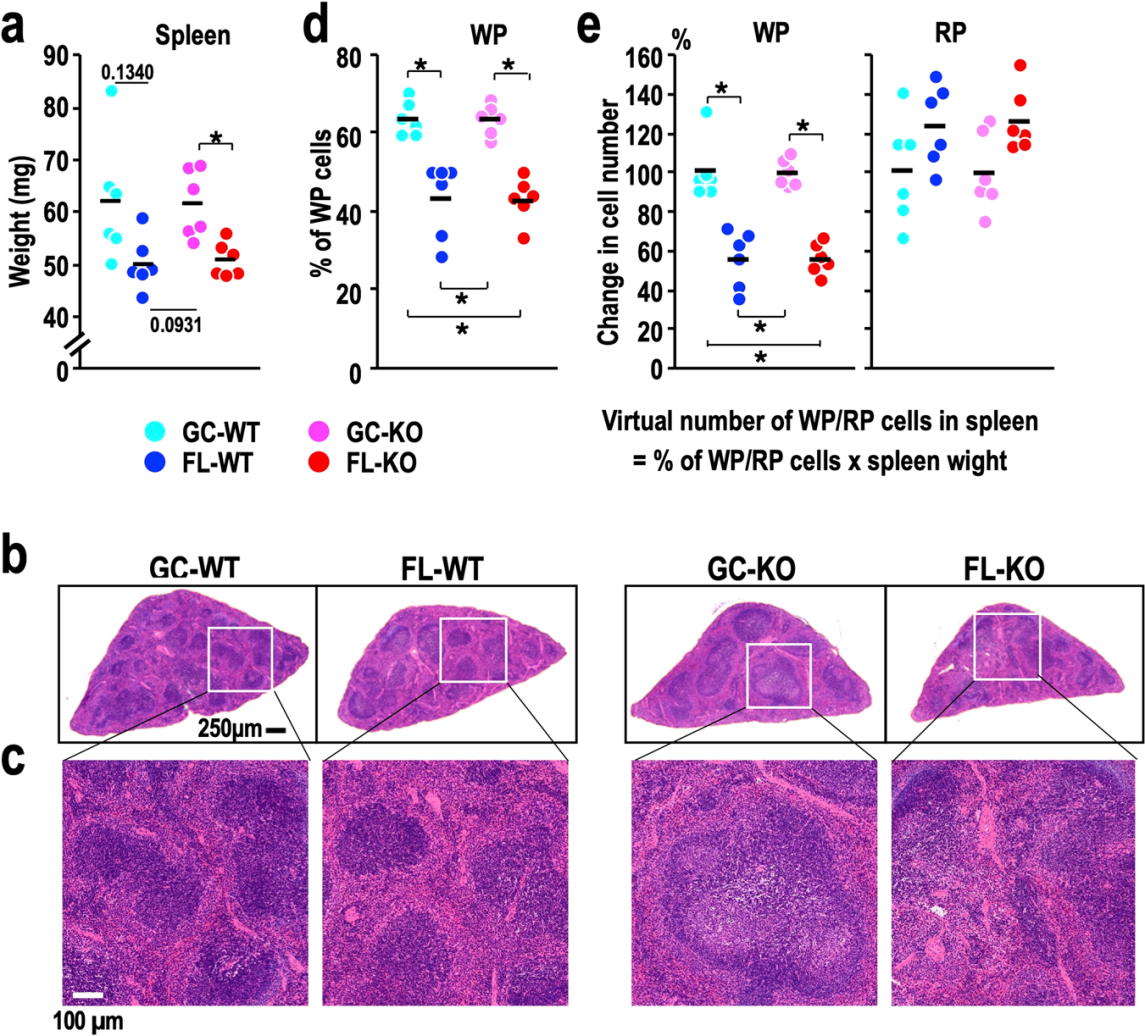
Changes in spleen size parameters. **a** Dot plot of spleen weight. Note that a reduction in spleen size becomes evident in the FL-KO mice. **b** Representative images showing HE staining of paraffin-embedded splenic cross sections from mice with the indicated genotypes. **c** Enlarged views of the selected regions (white boxes) from the images in (**b**). Note that shrinkage of the white pulp (WP) is obvious in FL-WT and FL-KO mice. **d** Frequency of WP cells in cross sections. **e** Calculated numbers of cells residing in WP (left) and red pulp (RP, right) areas in individual spleens. The mean value obtained for GC-WT mice in each group was set to 100%. Dots represent individual animals. Means are shown in the plots. *p < 0.05.

As RBC numbers and Hct values were increased by spaceflight, we next examined spleen mass in FL mice. Unexpectedly, spleen weight was decreased by spaceflight, and the decrease was significant between FL-KO mice and GC-KO mice (Fig. 4a). Consistent with this observation, a decrease in the ratio of spleen weight to total body weight after spaceflight was also observed^37^.

To address the reason for the decrease in spleen weight, we examined splenic cross sections by haematoxylin-eosin (HE) staining. We noticed a marked decline in white pulp (WP) areas compared with red pulp (RP) areas in the spleens of FL mice (Fig. 4b, c). Quantification of the numbers of WP and RP cells in the cross-sections was conducted by using QuPath software^38^. The results demonstrated that the percentage of WP cells was significantly decreased in the spleens of both FL-WT and FL-KO mice compared to those of the respective GC mice (Fig. 4d, Supplementary Fig. 7). We then calculated the numbers of WP and RP cells residing in individual spleens by multiplying the percentages of WP and RP cells obtained from the cross-sections by the spleen weight (Fig. 4e). We set the numbers of WP and RP cells in GC-WT mouse spleens as 100. The results indicate that the number of WP cells in the spleen was significantly decreased in FL mice, while the number of RP cells remained unchanged or increased. Thus, the decline in spleen weight appears to be due to the reduction in WP cells. Since WP cells consist largely of immune cells, including lymphocytes and macrophages, these results support our contention that spaceflight reduces the abundance of splenic immune cells, which leads to enhanced cytoprotection in space.

### Spaceflight has a strong impact on erythroid-gene expression in the spleen

**Fig. 5:**
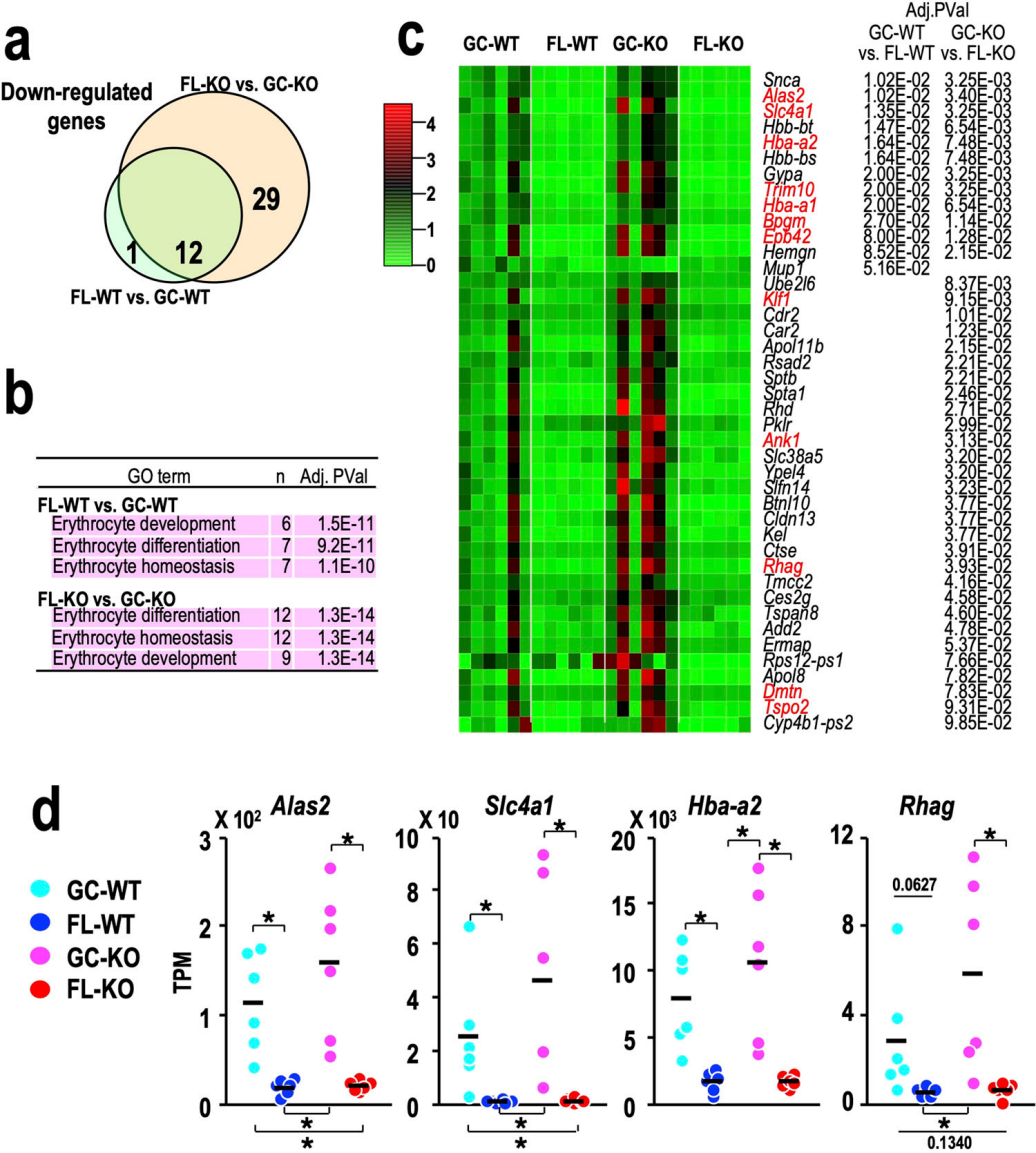
Genes downregulated in the spleen after spaceflight. **a** Venn diagram of downregulated differentially expressed genes (DEGs) in the spleen for the FL-WT vs. GC-WT (green) and FL-KO vs. GC-KO (orange) comparisons. **b** Gene Ontology (GO) analysis of downregulated DEGs for the FL-WT vs. GC-WT (upper rows) and FL-KO vs. GC-KO (lower rows) comparisons. GO terms related to erythropoiesis are marked with a pink background. The number of genes and the adjusted *p* values are shown. **c** Heatmap of the relative expression of downregulated genes in the FL-WT vs. GC-WT or FL-KO vs. GC-KO comparison. The mean value obtained for each gene in GC-WT mice was set to one. Erythroid-related genes are coloured in red. Adjusted *P* values for the FL-WT vs. GC-WT and FL-KO vs. GC-KO comparisons are shown. **d** Dot plot of the expression of representative erythroid-related genes. Dots represent individual animals. Means are shown in the plots. *p < 0.05.

We performed differentially expressed gene (DEG) analysis of splenic genes using integrated differential expression and pathway analysis (iDEP.95)^39^ based on the RNA-seq data obtained in our previous report^18^. We discovered that the expression of 13 genes was lower in FL-WT compared to GC-WT. Interestingly, the number of genes affected by the decrease due to spaceflight rose to 41 in the Nrf2-KO background (Fig. 5a). Most of the genes downregulated by spaceflight in WT mice overlapped with those downregulated in the Nrf2-KO background (12 out of 13; Fig. 5a), suggesting that the downregulation of splenic genes during spaceflight was extended if Nrf2 function was abrogated. Notably, consistent with a previous study^37^, the top three Gene Ontology (GO) terms associated with downregulated DEGs in FL-WT vs. GC-WT and FL-KO vs. GC-KO were all involved in “erythrocyte function” (Fig. 5b).

As shown in the heatmap of all 42 downregulated genes, the expression levels of the genes varied across individual GC-WT and GC-KO mice (Fig. 5c). In the heatmap, the mean value of gene expression for six GC-WT mice is set as 1, and erythrocyte genes are shown in red. While the expression of these 42 genes showed an increasing tendency in GC-KO mice compared with GC-WT mice, their expression was substantially decreased in both FL-WT and FL-KO mice. We confirmed that the expression of the *Alas2*, *Slc41a*, *Hba-a2* and *Rhag* genes decreased substantially after spaceflight, regardless of Nrf2 expression (Fig. 5d). These results thus demonstrate that space-related stress has a strong negative impact on the expression of erythroid-related genes in the spleen, which surpasses the contribution of Nrf2 to gene regulation.

### Immunophenotypic analyses of cryopreserved spleen cells by flow cytometry

**Fig. 6:**
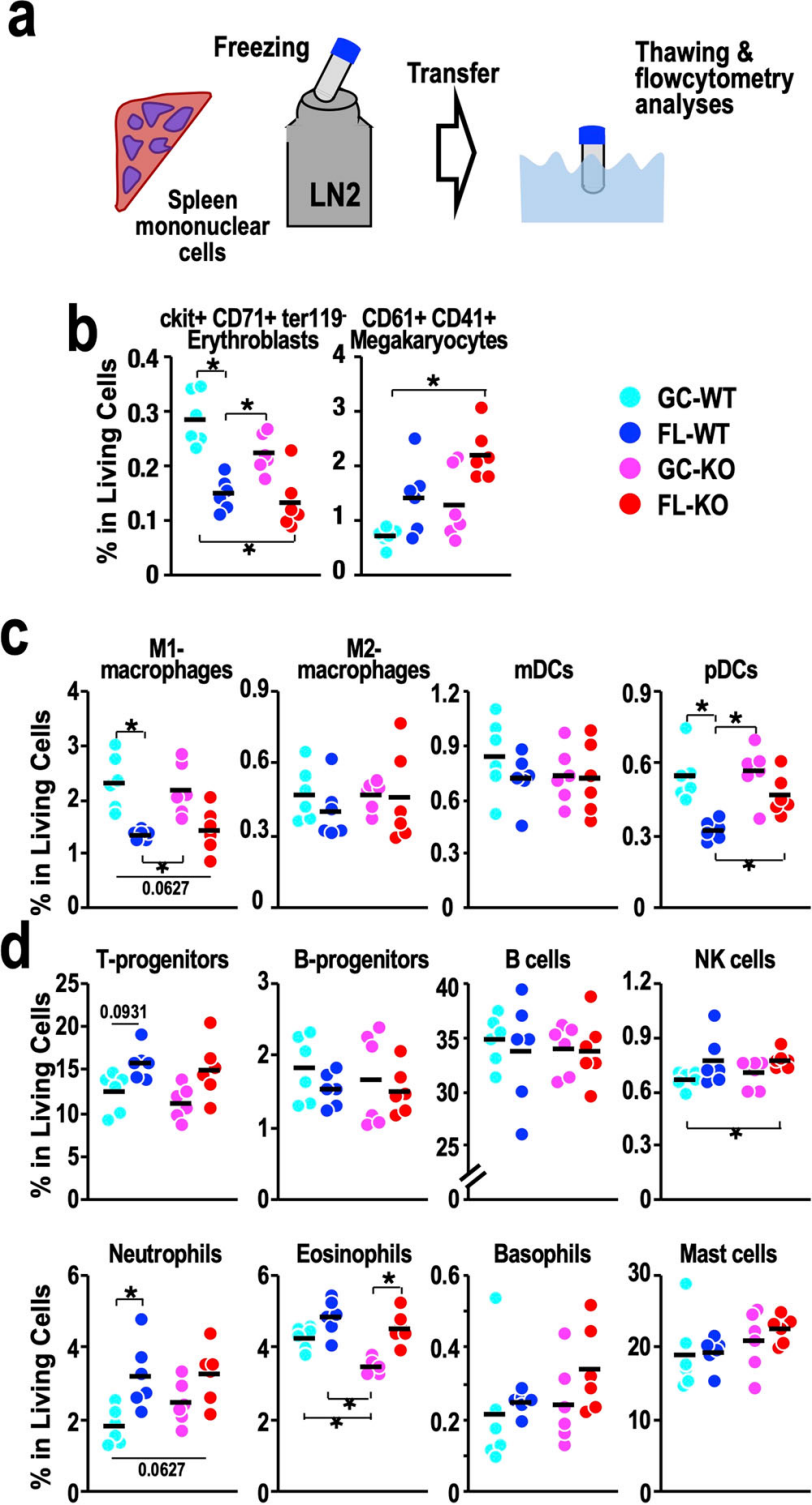
Populations of lineage-committed cells in the spleen. **a** Schematic diagram of flow cytometry analyses of spleen mononuclear cells. LN2, liquid nitrogen. **b** Dot plot of the frequencies of ckit^+^CD71^+^Ter119^-^ erythroblasts and CD61^+^CD41^+^ megakaryocytes in live cells from the spleen. **c** Dot plot of the frequencies of M1 and M2 macrophages, myeloid dendritic cells (mDCs) and plasmacytoid DCs (pDCs) in live cells from the spleen. **d** Dot plot of the frequencies of T-prognitors, B-progenitors, B cells, natural killer (NK) cells, neutrophils, eosinophils, basophils and mast cells in live cells from the spleen. Dots represent individual animals. Means are shown in the plots. *p < 0.05.

To examine the changes in haematopoietic cell populations induced by spaceflight, we performed flow cytometry analyses. While fresh samples are preferred for flow cytometry analysis, experimental limitations in the space mouse study did not allow us to perform sampling in the same place for FL and GC mice. Therefore, to address this issue, we decided to utilise cryopreserved samples that were thawed concomitantly (Fig. 6a).

We first measured the viability of the total and lineage-negative cells recovered from cryovials of splenic and bone marrow cells. We found that the viabilities of the fresh splenic and bone marrow cells were approximately 60% and 90%, respectively, and the viabilities of the cryopreserved cells varied across samples. The cryopreserved cells exhibited 50–90% viability compared to that of the fresh cells, with little difference between cryopreserved splenic and bone marrow cells (Supplementary Fig. 8a, b).

A surprising observation was that when we analysed the frequency of lineage-committed cells by surface marker expression, cells positive for Ter119, a marker for erythroid cells from the early proerythroblast to mature erythrocyte stages, showed very poor survival. The number of Ter119-positive cells was reduced to approximately 0% in all mice (Supplementary Fig. 8c). Thus, we excluded Ter119-positive cells from the experiment. Nonetheless, we found that cKit^+^CD71^+^Ter119^-^ erythroid progenitors and CD41^+^CD61^+^ megakaryocytes were viable (Supplementary Fig. 8d, e) and could be analysed. The abundance of cKit^+^CD71^+^Ter119^-^ erythroid progenitors was significantly reduced in both FL-WT and FL-KO mice (Fig. 6b). In contrast, the abundance of CD41^+^CD61^+^ megakaryocytes was increased in FL-WT and FL-KO mice, indicating that erythroid-megakaryocytic bifurcation is inclined towards the megakaryocytic linage in the spleens of mice in space.

Regarding immune-related cell populations, we found significant changes in the frequencies of two cell populations in the spleen. One was the M1 macrophage fraction (Fig. 6c), and the other was plasmacytoid dendritic cells (pDCs) (Fig. 6c). Both cell populations showed markedly decreased abundance after spaceflight. In contrast, we did not find substantial differences in the frequencies of M2 macrophages or myeloid DCs (mDCs) (Fig. 6c, middle two panels). Similarly, we did not find significant changes in the frequencies of lymphoid cells, granulocytes or mast cells in the spleen between the GC and FL groups, except for two cell populations (Fig. 6d). One such change was an increase in the abundance of neutrophils in FL-WT mice compared with GC-WT mice, and the other was an increase in eosinophil abundance in FL-KO mice compared with GC-KO mice (Fig. 6d). These results thus demonstrate that spaceflight alters the distribution of haematopoietic cell populations in the spleen within the Ter119-negative cell-gated fraction.

We then examined the frequencies of haematopoietic stem/progenitor cells (HSPCs) in the spleen, including lineage-negative/Sca1-positive/cKit-positive cells (LSKs), lineage-negative/Sca1-negative/cKit-positive cells (LKs), common myeloid progenitors (CMPs), granulocyte-macrophage progenitors (GMPs), megakaryocyte/erythrocyte progenitors (MEPs), long-term haematopoietic stem cells (LT-HSCs), short-term HSCs (ST-HSCs), multipotent progenitors subset 2 (MPP2s), multipotent progenitors subset 3 (MPP3s), and lymphoid-primed multipotent progenitors (LMPPs). These progenitor populations did not change substantially during spaceflight, except for LMPPs, whose abundance was moderately decreased in FL-WT mice compared with GC-WT mice (Supplementary Fig. 9a).

### Analyses of cryopreserved bone marrow cells by flow cytometry

**Fig. 7:**
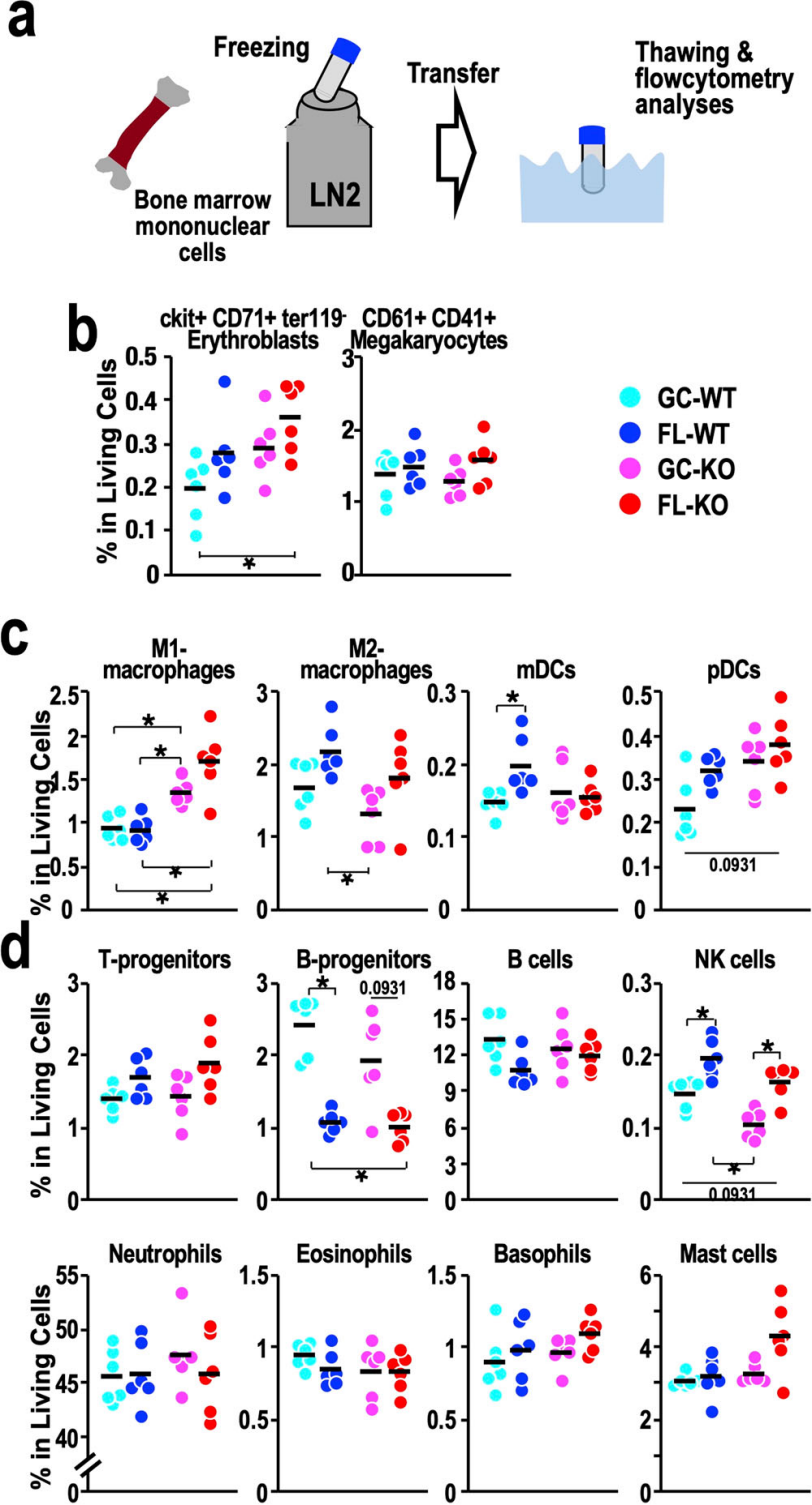
Populations of lineage-committed cells in the bone marrow. **a** Schematic diagram of flow cytometry analyses of bone marrow mononuclear cells. **b** Dot plot of the frequencies of ckit^+^CD71^+^Ter119^-^ erythroblasts and CD61^+^CD41^+^ megakaryocytes in live cells from the bone marrow. **c** Dot plot of the frequencies of M1- and M2 macrophages, mDCs and pDCs in live cells from the bone marrow. **d** Dot plot of the frequencies of T-progenitors, B-progenitors, B cells, NK cells, neutrophils, eosinophils, basophils and mast cells in live cells from the bone marrow. Dots represent individual animals. Means are shown in the plots. *p < 0.05.

We next performed similar flow cytometry analyses for frozen bone marrow samples (Fig. 7a). As shown in the previous section, the viability of the bone marrow cells exceeded that of the spleen cells (Supplementary Fig. 8a). As observed for the spleen samples, the frequencies of HSPCs in the bone marrow did not change substantially among the four mouse groups (Supplementary Fig. 9b), indicating that spaceflight does not influence the distribution of HSPCs.

We then analysed lineage-committed cells in the bone marrow and compared the results with those obtained for the spleen. Strikingly, we found that the frequencies of cKit^+^CD71^+^Ter119^-^ erythroid progenitors in the bone marrow were increased in FL-WT and FL-KO mice, while those of CD41^+^CD61^+^ megakaryocytes did not change substantially (Fig. 7b). These changes were completely different from those observed in the spleen (Fig. 6b).

Similarly, the distributions of immune-related cells were changed in the bone marrow after spaceflight, but the directions of the changes were quite different from those observed in the spleen. The frequencies of M1 macrophages did not change much after spaceflight. In contrast, Nrf2 deficiency strongly increased the frequency of M1 macrophages (Fig. 7c). The frequencies of mDCs were increased in FL-WT mice compared with GC-WT mice, while the frequencies of pDCs in FL-WT mice did not change much compared with those in GC-WT mice (Fig. 7c).

We also found a significant reduction in B-progenitor abundance and an increase in NK cell abundance in FL-WT and FL-KO mice compared with GC-WT and GC-KO mice, respectively, while the frequencies of T-progenitors, mature B cells, granulocytes and mast cells did not change much (Fig. 7d). Since the interaction between NK cells and B lymphocytes plays an important role in the immune system^40^, these changes may contribute, at least in part, to the impairment of the immune system during spaceflight. These results thus demonstrate that spaceflight indeed disrupts haematopoietic homoeostasis by affecting the distributions of cell populations, especially lineage-committed cell populations. This disruption is a complex phenomenon influenced by various regulatory cues, including the Nrf2 regulatory cascade.

### Nrf2 deficiency incites spaceflight-induced erythroid-gene suppression in the bone marrow

To address the molecular basis of these spaceflight-induced gene expression changes, we then performed RNA-seq analysis of bone marrow samples. To the best of our knowledge, this is the first study to examine gene expression in the bone marrow of animals after spaceflight. We also examined the influence of genetic Nrf2 depletion on gene expression in the bone marrow of mice after spaceflight.

**Fig. 8:**
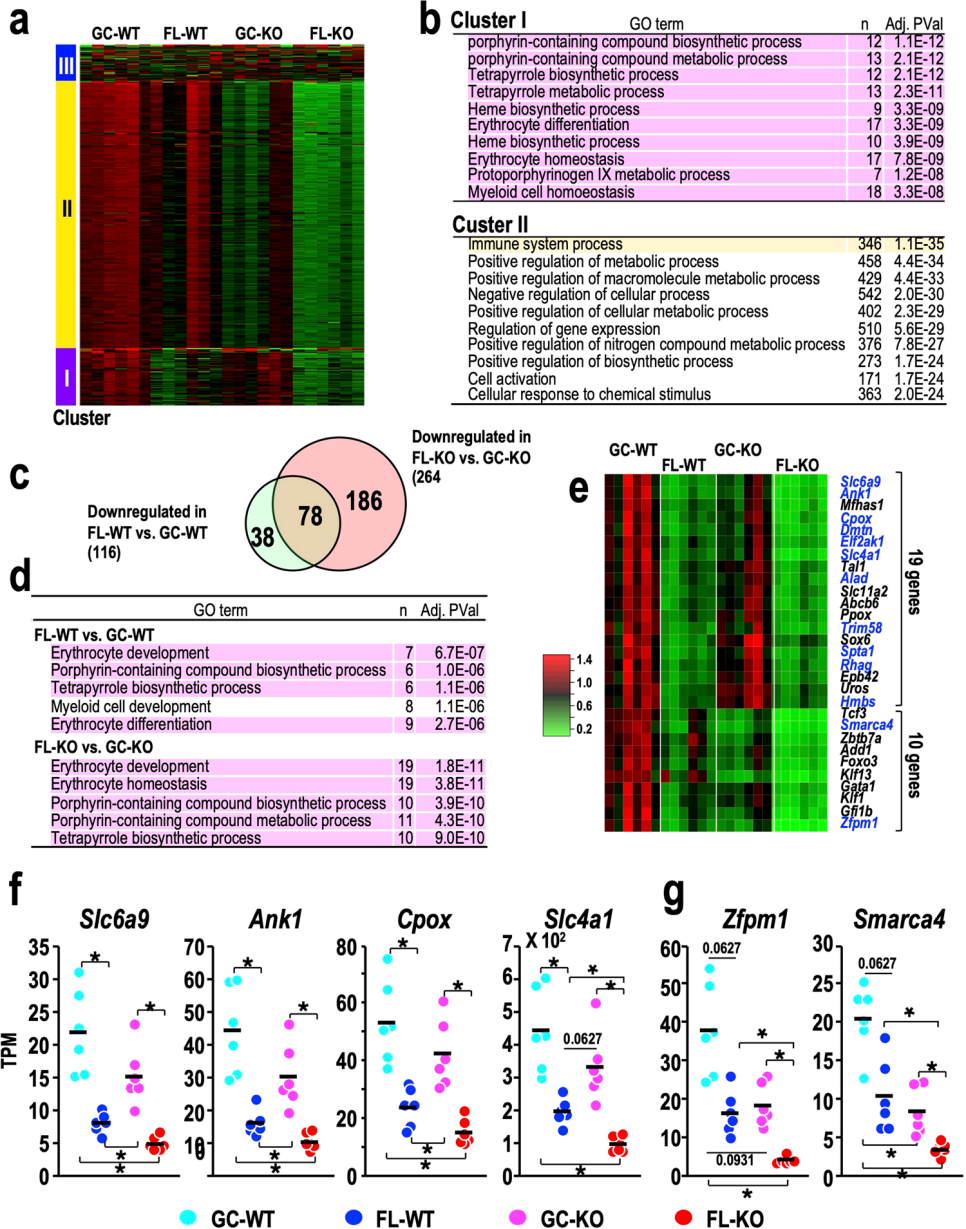
RNA-seq analysis of bone marrow samples focusing on the comparison between GC and FL mice. **a** Cluster analysis of DEGs based on the k-means method. **b** Cluster-specific GO enrichment analysis. GO terms related to erythropoiesis (marked with a pink background) and the immune system (marked with an orange background) are enriched in Clusters I and II, respectively. The number of genes and the adjusted *p* values are shown. **c** Venn diagram of downregulated DEGs in the bone marrow for the FL-WT vs. GC-WT (green) and FL-KO vs. GC-KO (orange) comparisons. **d** GO analysis of downregulated DEGs for the FL-WT vs. GC-WT (upper rows) and FL-KO vs. GC-KO (lower rows) comparisons. GO terms related to erythropoiesis are marked with a pink background. The number of genes and the adjusted *p* values are shown. **e** Heatmap of the relative expression of erythroid-related genes downregulated in comparisons of FL-WT vs. GC-WT (left two columns) and FL-KO vs. GC-KO (right two columns). The mean value obtained for each gene in GC-WT mice was set to one.　Downregulated DEGs in both the FL-WT vs. GC-WT and FL-KO vs. GC-KO comparisons are coloured blue. **f**, **g** Dot plot of the expression of representative erythroid-related genes. Dots represent individual animals. Means are shown in the plots. *p < 0.05.

We applied the top 2,000 genes that were differentially expressed among the four groups to the k-means algorithm of the iDEP.95 application and divided them into three groups (Fig. 8a). Cluster I included genes whose expression levels were almost equivalent in GC-WT and GC-KO mice as well as in FL-WT and FL-KO mice (*n* = 339). Cluster II included genes that were moderately downregulated in FL-WT mice and markedly downregulated in GC-KO mice compared with GC-WT mice, but this cluster of genes was even more downregulated in FL-KO mice than in GC-KO mice (*n* = 1465). Cluster III included genes without such specific characteristics (*n* = 196).

We then performed pathway analyses based on the data. An intriguing observation was that GO terms related to erythropoiesis were enriched *en bloc* in Cluster I (Fig. 8b), while a GO term corresponding to immune system process was most strongly enriched in Cluster II (Fig. 8b). No GO term was identified for Cluster III.

In parallel, we also conducted DEG analyses. We found 116 and 264 genes that were downregulated in the FL-WT vs. GC-WT and FL-KO vs. GC-KO comparisons, respectively (Fig. 8c). As 78 genes were downregulated in both comparisons, 38 and 186 genes were specifically downregulated in the FL-WT vs. GC-WT and FL-KO vs. GC-KO comparisons, respectively. GO analysis of the downregulated genes in FL-WT and FL-KO mice revealed that several subcategories related to erythropoiesis, including “erythrocyte development”, “porphyrin-containing compound biosynthetic process” and “tetrapyrrole biosynthetic process”, were associated with decreased expression in the FL groups, irrespective of whether the mice had the WT or Nrf2-KO background (Fig. 8d).

Among the DEGs downregulated by spaceflight, we found 29 genes that were categorised as erythropoiesis-related genes according to GO terms. When we conducted heatmap visualisation of the 29 genes, we found that Nrf2-KO reduced slightly the expression of 19 genes out of the 29 genes (Fig. 8e). For instance, spaceflight substantially decreased the expression of the *Slc6a9*, *Ank1*, *Cpox* and *Slc4a1* genes (Fig. 8f). In contrast, the absence of Nrf2 severely reduced the expression of the remaining 10 genes, and spaceflight further suppressed the expression of these genes (Fig. 8e). For instance, the absence of Nrf2 strongly decreased the expression of the *Zfpm1* and *Smarca4* genes, and spaceflight exacerbated these decreases (Fig. 8g).

Upon comparing erythroid-gene expression between WT and Nrf2-KO mice under GC and FL conditions separately, we observed that the reduction of erythroid genes in FL condition was amplified by the Nrf2-KO status. This observation was made irrespective of the influence of Nrf2-KO on erythroid-gene expression in ground conditions (Supplementary Fig. 10). Taken together, these results demonstrate that spaceflight suppressed the expression of a group of erythroid-related genes and that Nrf2 deficiency amplified the spaceflight-induced erythroid-gene suppression in the bone marrow.

In contrast, spaceflight-induced upregulation of gene expression was quite rare. Through closer inspection, we found 7 genes that were upregulated in FL-WT mice compared with GC-WT mice, including three different variants of *Stfa* genes encoding Stefin A1, A2 and A3 (Supplementary Fig. 11). Stefin A acts as a cysteine protease inhibitor and plays important roles in many biological processes, such as the immune response, inflammation^41^ and platelet-dependent thrombus formation^42^. This imbalance in proteolytic activity might be involved in the pathophysiological changes caused by spaceflight.

### Nrf2 deficiency exacerbates spaceflight-induced immunosuppression

**Fig. 9:**
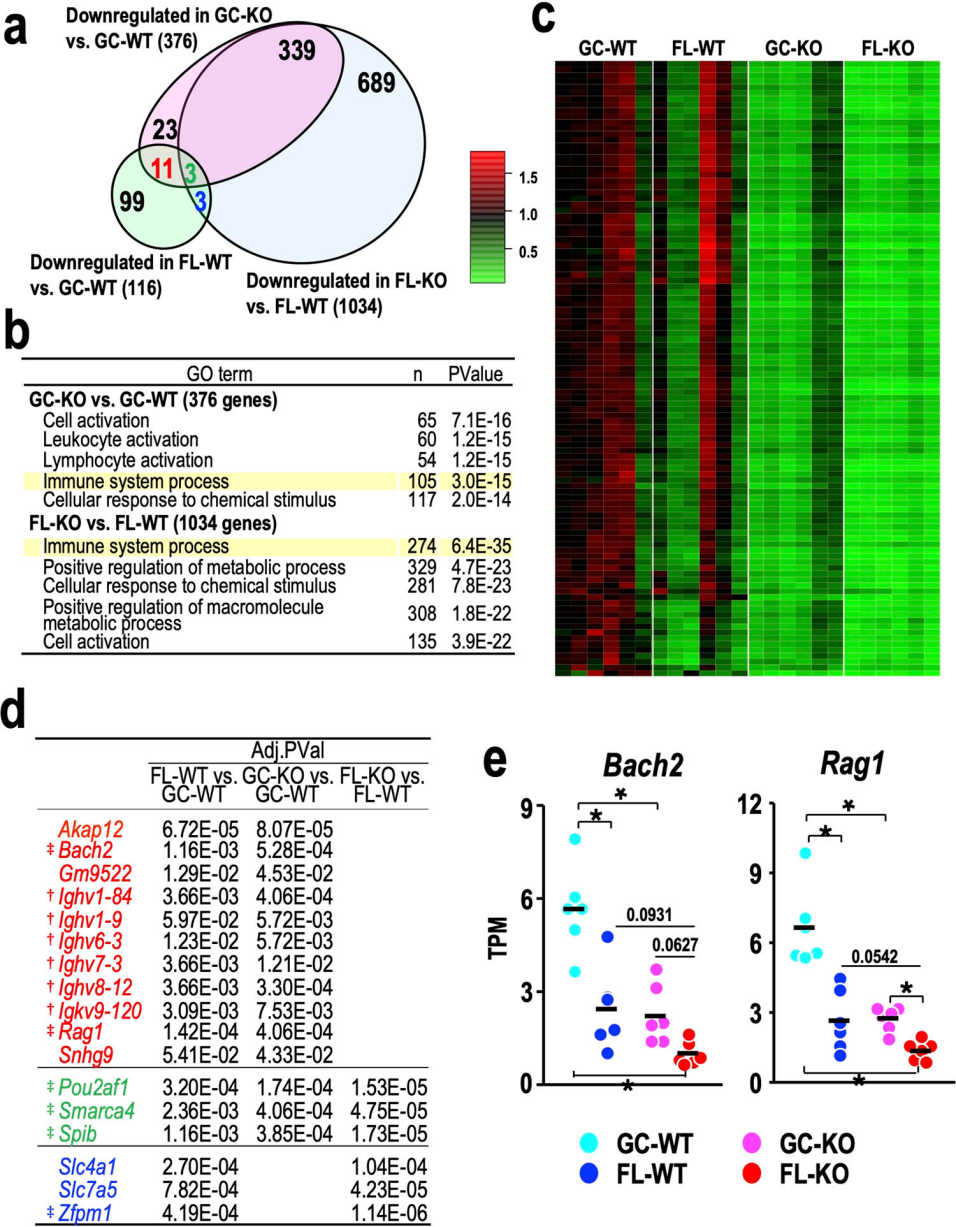
RNA-seq analysis of bone marrow samples focusing on the comparison between WT and Nrf2-KO mice. **a** Venn diagram of three sets of downregulated DEGs from the GC-KO vs. GC-WT (pink), FL-KO vs. FL-WT (blue) and FL-WT vs. GC-WT (green) comparisons. The numbers of genes in each section are shown. The green number represents the number of overlapping DEGs among all three comparisons. The number of downregulated genes in the FL-WT vs. GC-WT comparison that overlap with those in the GC-KO vs. GC-WT (pink) and FL-KO vs. FL-WT (blue) comparisons, excluding DEGs in the triple overlapping region, are indicated by red and blue, respectively. **b** GO analysis of downregulated DEGs from the GC-KO vs. GC-WT (upper rows) and FL-KO vs. FL-WT (lower rows) comparisons. The number of genes and the adjusted *p* values are shown. **c** Heatmap of the relative expression of 105 genes categorised as “immune system process” among the downregulated DEGs from the GC-KO vs. GC-WT comparison. **d** Overlapping downregulated DEGs with adjusted *P* values for the FL-WT vs. GC-WT, GC-KO vs. GC-WT, and FL-KO vs. FL-WT comparisons. Red, blue and green correspond to the genes in (**a**). Daggers indicate genes encoding immunoglobulin, and double daggers indicate genes encoding factors related to immune system processes. **e** Dot plot of the expression levels of the *Back2* and *Rag1* genes. Dots represent individual animals. Means are shown in the plots. *p < 0.05.

We next wanted to investigate how Nrf2 depletion influences gene expression profiles in bone marrow cells from spaceflight mice. To this end, we performed two sets of DEG analyses utilising genes downregulated in the bone marrow of Nrf2-KO mice: GC-KO vs. GC-WT and FL-KO vs. FL-WT. We found that 376 and 1034 genes were decreased due to Nrf2-KO under GC and FL conditions, respectively (Fig. 9a). The number of genes downregulated by Nrf2-KO was much larger than that downregulated by spaceflight, which was 116 (Fig. 9a).

Of note, 342 out of 376 genes that were decreased in the Nrf2-KO mice under GC conditions were concomitantly downregulated in the FL-KO mice, indicating that the genes downregulated due to Nrf2-KO under GC conditions were almost all involved in the gene cluster of 1034 downregulated genes under FL conditions. This result demonstrates that genetic Nrf2 depletion more severely affects bone marrow homoeostasis under spaceflight conditions than under ground conditions and supports our contention that Nrf2 plays important roles in the protection of homoeostasis during spaceflight.

We found that the GO term “immune system process” was significantly enriched among the downregulated DEGs in both the GC-KO vs. GC-WT and FL-KO vs. FL-WT comparisons (Fig. 9b). It should be noted that this GO term was the best hit in the comparison of spaceflight conditions. Closer inspection of the heatmap showing that immune system process-related genes decreased in GC-KO mice (i.e., 105 DEGs) revealed that these genes also showed decreased expression in FL-WT mice, and these decreases were reproduced in FL-KO mice (Fig. 9c). These results suggest that spaceflight conditions have a similar effect to Nrf2-KO conditions.

The finding that both Nrf2-KO and spaceflight conditions elicited a decrease in immune system process-related genes prompted us to examine the details of these changes, including whether these changes lead to immune suppression or activation. To address this point, we superimposed 116 downregulated DEGs identified in the FL-WT vs. GC-WT comparison with those downregulated in both the GC-KO vs. GC-WT and FL-KO vs. FL-WT comparisons. As shown in Fig. 9a, a total of 17 out of the 116 genes overlapped with those downregulated in the comparisons of GC-KO vs. GC-WT and/or FL-KO vs. FL-WT. Among these 17 genes, 6 were immunoglobulin genes, and 6 were genes belonging to the immune system process signature (Fig. 9d). For instance, spaceflight and Nrf2-KO independently and additively induced a decrease in the expression of the *Bach2* and *Rag1* genes, whose products are known to play important roles in lymphoid development^43,44^ (Fig. 9e). Thus, spaceflight decreases and concomitant Nrf2-KO exacerbates the expression of immune genes in the bone marrow, leading to immunosuppression.

### Differential contributions of spaceflight to Nrf2 target gene expression in the spleen and bone marrow

**Fig. 10:**
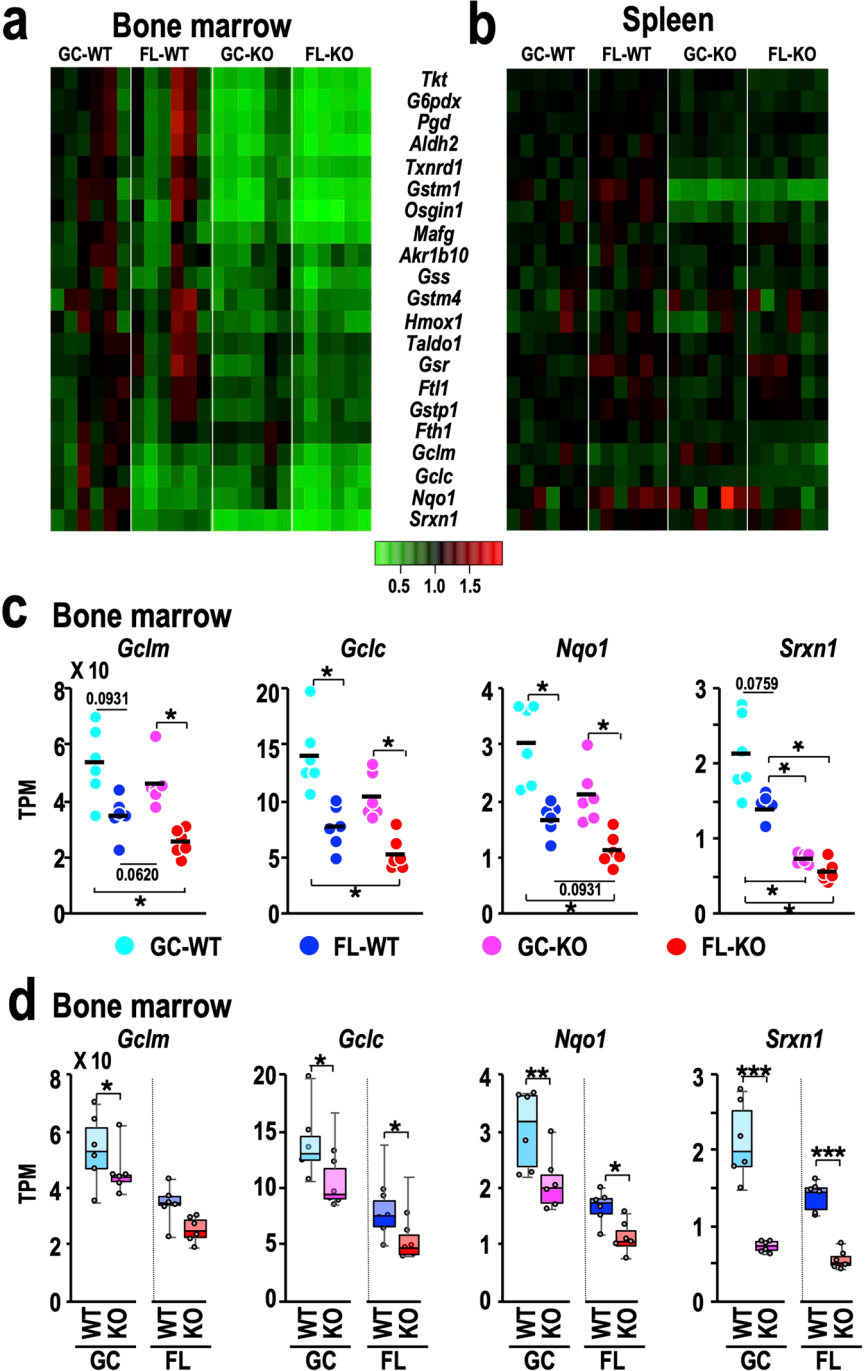
Changes in the expression of Nrf2 target genes. **a, b** Heatmap of the relative expression of Nrf2 target genes in the bone marrow (**a**) and spleen (**b**) from GC-WT, FL-WT, GC-KO and FL-KO mice. The mean value of each gene in GC-WT mice was set to one in each panel. **c** Dot plot of the expression of the *Gclm*, *Gclc, Nqo1* and *Srxn1* genes in the bone marrow. Dots represent individual animals. Means are shown in the plots. *p < 0.05. **d** Box-and-dot plots of the expression of *Sclm*, *Gclc*, *Nqo1* and *Smarca4* genes in the bone marrows. One-sided Wilcoxon rank sum tests were performed to evaluate the differences between Nrf2-KO and control WT mice in the ground (left part in each panel) and spaceflight (right part) conditions. *P < 0.05.

It has been shown that spaceflight activates the expression of Nrf2 target genes in various tissues and organs^18,20^. To address the question of whether this effect of spaceflight also occurs in the bone marrow and spleen, we compared the expression profiles of typical Nrf2 target genes in the four groups of mice, *i.e*., GC-WT, FL-WT, GC-KO and FL-KO. Interestingly, we found that the expression of Nrf2 target genes, which was found to be mostly increased in other tissues^18^, was not activated but rather decreased in the bone marrow of FL-WT mice compared with GC-WT mice (Fig. 10a). The expression of these Nrf2 target genes was strongly decreased in GC-KO and FL-KO mice compared with GC-WT and FL-WT mice (Fig. 10a). In stark contrast, the expression of Nrf2 target genes in the spleen was comparable in the four groups (Fig. 10b), indicating that neither Nrf2-KO nor spaceflight substantially influenced the expression of Nrf2 target genes in the spleen. These findings imply that while in most tissues, spaceflight induces Nrf2 activity and Nrf2 protects against spaceflight-elicited perturbations in homoeostasis, in the bone marrow, Nrf2 activation by spaceflight is rather weak and cannot fully maintain homoeostasis. In the spleen, the contribution of Nrf2 is limited even under basal conditions.

We found four genes whose expression was significantly reduced in FL-WT mice compared with GC-WT mice (Fig. 10c). The products of the *Gclm*, *Gclc*, *Nqo1* and *Srxn* genes are all known to play important roles in the redox regulation pathway^45,46^. These findings demonstrate that the bone marrow is quite sensitive to spaceflight-related stresses. Strikingly, while spaceflight substantially decreased the expression of the *Gclm*, *Gclc*, *Nqo1* and *Srxn* genes, the concomitant absence of endogenous Nrf2 further decreased expressions of those genes (Fig. 10c, d). While bone marrow cells can be partially protected by the increase in endogenous Nrf2 activity, the magnitude of Nrf2 induction by spaceflight per se appears not to be sufficient, so hematopoietic and immune suppression phenotypes become evident, and Nrf2 inducers may help to protect humans during space travel.

## Discussion

In this study, we conducted haematological and immunological assessments of the mice from the MHU-3 mission, which sent six Nrf2-KO mice and six WT mice to space. To overcome difficulties inherent to the space study, we have incorporated several experimental challenges into this study. As a result, we found that Nrf2 does not influence much the protection of erythropoiesis during spaceflight. In contrast, Nrf2 plays important protective roles in the immune system during spaceflight. In fact, spaceflight-induced suppression of immune-related gene expression is markedly enhanced by genetic depletion of Nrf2, while spaceflight-induced reductions in erythroid-gene expression are significant in the spleen and bone marrow, regardless of the Nrf2 expression level. These findings thus indicate that Nrf2 contributes to the maintenance of the immune system during spaceflight and suggest that improving the level of baseline Nrf2 activity alleviates the degree of immune suppression during spaceflight. We surmise that this finding is particularly important for further exploration of space beyond the ISS. If we consider upcoming plans to develop space travel, cosmic radiation in the ISS is mild, and a one-month stay in the ISS is short. Therefore, we believe that the importance of Nrf2 activation during space travel should be studied further and in more detail.

In this study, we showed that spaceflight has the potential to increase the risk of thrombotic microangiopathy, accompanied by disturbance of the coagulation and fibrinolysis system. Since internal jugular venous thrombosis was identified as a space travel-associated disease^47^, coagulation regulation during spaceflight has been a concern^48–50^. In this study, we found increases in MPV and PDW in FL mice. Although the platelet number remained unchanged, it is conceivable that the increases in MPV and PDW represent an accelerated platelet turnover rate. We also observed an increasing trend for *vWF* expression in various tissues of FL mice. vWF is a well-known factor that plays central roles in thrombosis caused by inflammation^34,51^. Spaceflight-induced activation of *vWF* gene expression appeared to be accelerated in combination with the Nrf2-KO background, implying that Nrf2 is involved in protection against endothelial inflammation and injuries^52^.

Elevated expression of genes involved in the congealing-fibrinogenolytic pathway is also observed in the liver in FL mice, accompanied by elevated expression of *Stfa* genes, a hallmark of platelet activation, in the bone marrow^42^. To the best of our knowledge, these are the first analyses of the coagulation and fibrinolysis system in mice after spaceflight, and we envisage that intravascular coagulation is activated by spaceflight, and consequently, the expression of genes encoding the coagulation and fibrinogenolytic proteins is elevated to compensate for their consumption^53^. An important point here is that the spaceflight-induced changes in the congealing-fibrinogenolytic pathway are enhanced by Nrf2-KO, suggesting that Nrf2 contributes, at least in part, to protecting intravascular thrombosis during spaceflight.

The elevation of Hct values in astronauts during the initial phase of space travel has been considered to be due to reduced plasma volume caused by adjustment of the body fluid balance to microgravity^54^. While elevated Hct values are found during the early period of a stay in the space shuttle^9^, polycythemia is gradually reversed through haematopoietic adaptation to microgravity^11^, accompanied by increased haemolysis^10,12^. Upon returning to normal gravity, a redistribution of body fluids occurs, along with an increase in circulating blood flow, which causes postflight anaemia^9^. Therefore, astronauts with a longer stay in microgravity develop more severe anaemia^12^. In stark contrast, mice do not show polycythemia at L + 18 in the ISS, but instead, a marked increase in RBC mass is found after landing. We surmise that the following differences between mice and humans may be pertinent to this discrepancy. One is that the life span of mouse erythrocytes is approximately 40 days, which is 1/3 shorter than that of human erythrocytes^55^; thus, mice may complete erythropoietic adaptation faster than humans during their stay in microgravity. The other is that the impact of microgravity on Hct values may be different between bipedal and quadruped animals.

Through a series of the MHU-3 studies, we discovered that Nrf2 plays pivotal roles in maintaining lipid metabolism homoeostasis in response to spaceflight stress^8,20,21^. Considering that lipid serves as a fuel source for myelopoiesis in bone marrow^56^, it is plausible to hypothesise that the immunosuppression induced by spaceflight and Nrf2 deficiency may be mediated by disturbed lipid metabolism.

This study has clarified that spaceflight affects both the erythroid and immune systems in the spleen and bone marrow, but the influences appear to be distinct between these two organs. In mice, the spleen is a haematopoietic organ that plays critical roles in stress-erythropoiesis, while the bone marrow exhibits balanced haematopoiesis under both steady-state and stress conditions. In contrast, the bone marrow is the only bona fide haematopoietic organ in humans. Consistent with preceding studies^37,57^, we found in this study that the size of the spleen decreases in FL mice. Closer examination of the spleen in FL mice further revealed that the white pulp was significantly reduced. Since the white pulp is the site where myeloid and lymphoid cells reside, this observation further supports the notion that spaceflight induces immunosuppression. Analyses of Nrf2-KO mice revealed that Nrf2 knockout does not influence the expression of Nrf2 target genes in the spleen of FL or GC mice.

In contrast, we found that the size of the red pulp did not change much after spaceflight. In this context, the expression of GATA1 and related erythroid genes is downregulated after spaceflight^37^. One plausible explanation for these observations is that while humans develop postflight anaemia, mice develop polycythemia. Therefore, in response to the increase in RBC numbers, the expression of GATA1 and erythroid genes may be decreased. Alternatively, changes in erythroid-gene expression might occur during the transition from microgravity to normogravity, explaining why the size of the red pulp did not change much at the time of analysis.

## Methods

### Animals

All animal experiments were approved by the Institutional Animal Care and Use Committees of JAXA (protocol numbers 017-001 and 017-014), NASA (protocol number FLT- 17-112), Explora BioLabs (EB15-010C), and Tohoku University (2017MdA-328) and conducted according to the related guidelines and applicable laws of Japan and the United States of America. Nrf2-KO and WT male mice provided for the MHU-3 project and GC experiments were selected after 3-weeks acclimation in individual housing cages, based on the health condition^18,21^. Blood collection from the distal end of the tail was performed with tail clippers (KAI, PQ3357)^18,21^. Haematopoietic indices of blood collected from IVC were measured with VetScan HM5 autohemocytometers (Abaxis) on the US West Coast and Celltac-α MEK6450 autohemocytometers (Nihon Koden) in Japan. Bone marrow cells collected from the right pelvis and tibia were suspended in phosphate-buffered saline (PBS). Spleen mononuclear cell suspensions were isolated by filtration through a 100-μm cell strainer (Corning) to remove tissue fragments. After washing with PBS once, the cell pellets were resuspended in CELLBANKER2 (ZENOAQ RESORCE). After the samples were divided into three tubes, each cell suspension aliquot was frozen on dry ice and cryopreserved in liquid nitrogen.

### Quantification of cells in the spleen

Each spleen was divided transversely into three pieces, and a centre piece was fixed in Mildform 10 N (Wako), followed by embedding in paraffin. Two 3-µm-thick sections were prepared from each sample and stained with haematoxylin-eosin. Images were scanned using SLIDEVIEW VS200 (Olympus) and processed using QuPath software (v.0.3.2)^38^. To quantify WP and RP cells, an algorithm for cell detection was used in QuPath’s built-in cell detection programme, with representative pictures in which WP and RP areas were selected manually, and then the cell classification programme of QuPath was performed following the algorithm.

### Flow cytometry

The cryopreserved cells were rapidly thawed at 37 °C and immediately transferred into high-glucose DMEM (Nacalai) with 20% foetal bovine serum (FBS). After washing once with medium, the cells were washed once more with PBS. Lineage depletion was performed using a cocktail of biotinylated antibodies against Ter119, B220, Gr1, CD8, CD4, CD11b and CD127, each at a concentration of 1.0 μg/mL, followed by removal using Dynabeads M-280 streptavidin-conjugated magnetic beads (Thermo Fisher Scientific). Cells were labelled with fluorescence labelled antibodies at a concentration of 1.0 μg/mL and incubated on ice in the dark for 20 minutes. Subsequently the cells underwent two washes with PBS and were then resuspended in PBS containing 2% FBS. The debris exclusion was accomplished by creating a plot depicting the relationship between forward scatter (FSC)-area (A) vs. the side scatter (SSC)-A. To exclude doublet cells, a graph depicting FSC-height (H) against FSC-width (W) was employed, followed by another graph showing SSC-H vs. the SSC-W. The exclusion of dead cells was achieved through the utilisation of 7-Amino-Actinomycin D (BD Biosciences). The details of the fluorescently labelled antibodies used for flow cytometry analyses are shown in Supplementary Table 1 and gating strategies are provided in Supplementary Methods. The stained cells were filtered through a 35-μm strainer cap (FALCON) and analysed with BD FACSAria II and BD FACSDiva software (Becton Dickinson).

### RNA-sequence analysis

Total RNA was isolated from the cryopreserved bone marrow cells in CELLBANKER2 liquid by directly adding 3 mL ISOGEN-LS (NIPPON GENE) at room temperature. RNA integrity was assessed using an Agilent 2200 TapeStation. Two hundred nanograms of total RNA was subjected to cDNA library preparation using the MGIEasy RNA Directional Library Prep Set (MGI). The libraries were sequenced using DNBSEQ-G400 (MGI). The raw reads were mapped to the mouse mm10 genome using STAR (version 2.6.1)^58^. Transcripts per million (TPM) values were obtained to measure gene expression using RSEM (version 1.3.1)^59^. TPM values of spleen samples were obtained from a previously published dataset^18^. The TPM was normalised in iDEP.95^39^. Genes with a TPM above one in at least one sample were included in further analyses. k-means clustering was performed by iDEP.95. DEGs were analysed with a false discovery rate cut-off <0.1 and a fold change cut-off ≥2 using iDEP.95. Global analysis heatmaps were produced using R-based heatmap.3 and gplots packages.

### Statistics and reproducibility

Data points represent biological replicates. To assess dissimilarities among GC-WT, FL-WT, GC-KO and FL-KO groups, The Kruskal‒Wallis tests were performed. In cases where the *p*-value was less than 0.05, further Steel-Dwass multiple comparisons were conducted to evaluate differences among all four groups. For comparing the time-course changes in haematocrit values, a two-tailed Wilcoxon signed-rank test was employed in a pairwise manner. To assess the disparities between Nrf2-KO and control WT mice under both ground and spaceflight conditions, one-sided Wilcoxon rank sum tests were independently performed. Statistical analysis was carried out using JMP Pro 16 software (SAS Institute).

### Reporting summary

Further information on research design is available in the Nature Portfolio Reporting Summary linked to this article.

### Supplementary information

Supplementary information

Description of Additional Supplementary Files

Supplementary Data 1

Reporting Summary

## Data Availability

The data depicted in this publication have been deposited in NCBI’s Gene Expression Omnibus^60^ and accessible through GEO Series accession number (GSE240654). Supplementary Data 1 includes the source data corresponding to the graphs presented in the main figures and supplemental figures. All relevant data are available from the corresponding authors upon reasonable request.
