# Supplementary material for: Nrf2 alleviates spaceflight-induced immunosuppression and thrombotic microangiopathy in mice
Source: Commun Biol. 2023 Aug 25;6:875. doi: 10.1038/s42003-023-05251-w (PMC10457343; doi:10.1038/s42003-023-05251-w)
Supplement: Supplementary file 1 — Supplementary information [file 42003_2023_5251_MOESM1_ESM.pdf]

Supplementary Information

Supplementary Table 1

Antibodies used for flow cytometry analyses.

Supplementary Methods

Gating strategies used for flow cytometry analyses.

Supplementary Fig. 1

Pictures of the centrifuge tubes used to measure t-Hct.

Supplementary Fig. 2

Trimethylamine N-oxide levels are elevated in the plasma during spaceflight.

Supplementary Fig.3

Comparison of platelet parameters between Nrf2-KO and wild-type control mice in the ground and spaceflight conditions.

Supplementary Fig. 4

Comparison of *vWF* gene expression between Nrf2-KO and wild-type control mice in the ground and spaceflight conditions.

Supplementary Fig. 5

Gene expression changes for tissue inflammation markers.

Supplementary Fig. 6

Comparison of coagulation and fibrinolysis gene expression between Nrf2-KO and wild-type control mice in the ground and spaceflight conditions.

Supplementary Fig. 7

Original and colorized images of HE staining showing WP and RP cell detection, and classification.

Supplementary Fig. 8

Flow cytometry analyses evaluating cell viability in cryopreserved samples.

Supplementary Fig. 9

Populations of haematopoietic stem and progenitor cells in the spleen and bone marrow of mice after spaceflight.

Supplementary Fig. 10

Comparison of erythroid-related gene expression between Nrf2-KO and wild-type control mice in the ground and spaceflight conditions.

Supplementary Fig. 11

Expression of *Stfa* gene variants in the bone marrow.

Supplementary Table 1

Antibodies used for flow cytometry analyses.

	Antigen	Label	Manufacturer	LSK	LT-HSC	ST-HSC	MPP2	MPP3	LMPP	LK	CMP	GMP	MEP
HSPC	ckit	APC-eF780	eBioscience	+	+	+	+	+	+	+	+	+	+
	Sca1	BV421	BioLegend	+	+	+	+	+	+	-	-	-	-
	CD150	BV510	BioLegend		+	-	+	-	-				
	CD48	FITC	BioLegend		-	-	+	+	+				
	FLT3	PE	BioLegend		-	-	-	-	+				
	CD34	Alexa Fluor 64	BioLegend								mid	low~mid	low~mid
	CD16/32	PE-Cy7	BioLegend								mid	high	low

	Antigen	Label	Manufacturer	M1-MΦ	M2-MΦ	Baso	Neutro	Eosino	Mast	NK	mDC	pDC
Lineage Commited cells (1)	CD11b	FITC	BioLegend	+	+		+	-		-	+	-
	F4/80	APC	BioLegend	+	+					-	-	-
	Gr-1	APC-eF780	BioLegend	-	-	+	+	+	-	-		+
	FcεR1	PE	BioLegend			+	-	-	+	-	-	-
	CD11c	PE-Cy7	BioLegend	+	-	-	-	-	-	-	+	+
	NK1.1	BV421	BD Biosciences	-	-	-	-	-	-	+	-	-

	Antigen	Label	Manufacturer	T-pro	B-pro	B	Ery	Meg
Lineage Commited cells (2)	ckit	APC-eF780	eBioscience				+	
	IL7Ra	PE-Cy7	eBioscience	+	+	-	-	-
	CD19	BV510	BD Biosciences	-	+	+	-	-
	Ter119	APC	BD Biosciences	-	-	-	-	-
	CD71	BV421	BioLegend	-	-	-	+	-
	CD41	FITC	eBioscience	-	-	-	-	+
	CD61	PE	BD Biosciences	-	-	-	-	+

	Antigen	Label	Manufacturer
Leneage depletion	Ter119	biotinylated	BioLegend
	B220	biotinylated	BioLegend
	Gr1	biotinylated	BioLegend
	CD8	biotinylated	BioLegend
	CD4	biotinylated	eBioscience
	CD11b	biotinylated	eBioscience
	CD127	biotinylated	eBioscience

APC: Allophycocyanin
APC-eF780: APC-eFluor 780
BV421: Brilliant Violet 421
BV510: Brilliant Violet 510
FITC: Fluorescein isothiocyanate
PE: Phycoerythrin
PE-Cy7: PE-Cyanin 7

Supplementary Methods

Gating strategy for HSPCs.

For the LSK and LK populations, two-dimensional dot plots were generated to analyse the expression of ckit and Sca1 in the lineage-negative cell population. Subsequently, in the LSK population, after excluding FLT3-positive cells, two-dimensional dot plots of CD150 and CD48 expression were constructed to investigate the LT-HSC, ST-HSC, MPP2, and MPP3 populations. For the LMPP population, FLT3-positive cells were gated from the LSK population, and two-dimensional dot plots of CD150 and CD48 expression were constructed. To examine the CMP, GMP, and MEP populations, two-dimensional dot plots of CD34 and CD16/32 expression were generated within the gating fraction of LK cells.

Gating strategy for M1- and M2 macrophages, basophils, neutrophils, eosinophils, mast cells, NK cells, mDCs and pDCs.

To characterize the M1- and M2-macrophage populations, double-negative cells for Gr-1 and NK1.1 expression were initially gated, and the double-positive population of CD11b and F4/80 expression was gated. Subsequently, we generated one-dimensional histograms to analyse CD11c expression. For basophil and mast cell populations, after gating double-negative cells for CD11c and NK1.1 expression, two-dimensional dot plots of Gr-1 and FcεR1 expression was created. For neutrophil and eosinophil populations, FcεR1negative cells were gated from double-negative populations of CD11c and NK1.1 expression, and then two-dimensional dot plots of CD11b and Gr-1 expression were generated. In the analysis of NK cell population, CD11c-negative and NK1.1-positive cells were gated from the double-negative population of F4/80 and FcεR1 expression. Subsequently, two-dimensional dot plots of CD11b and Gr-1 expression were constructed. For mDC and pDC populations, we gated CD11c-positive and NK1.1-negative cells from the double-negative population of F4/80 and FcεR1 expression. Then, two-dimensional dot plots were created to analyse CD11b and Gr-1 expression.

Gating strategy for T-progenitors, B-progenitors, B cell, erythroblasts, megakaryocytes.

For T-progenitor, B-progenitor and B cell populations, double-negative cells for CD41 and CD61 expression were gated from the double-negative populations based on CD71 and Ter119 expression. Subsequently, we constructed two-dimensional dot plots to examine the expression of IL7Ra and CD19. In the case of erythroblast population, double-negative cells for CD41 and CD61 expression were gated from double-negative population of IL7Ra and CD19 expression. Furthermore, we also excluded cells with positive for Ter119 expression. Subsequently, we generated two-dimensional dot plots to analyse the expression of ckit and CD71. For the Megakaryocyte population, double-negative cells for CD71 and Ter119 expression were gated from double-negative populations of IL7Ra and CD19 expression, followed by the creation of two-dimensional dot plots to assess CD41 and CD61 expression.

Supplementary Fig. 1.

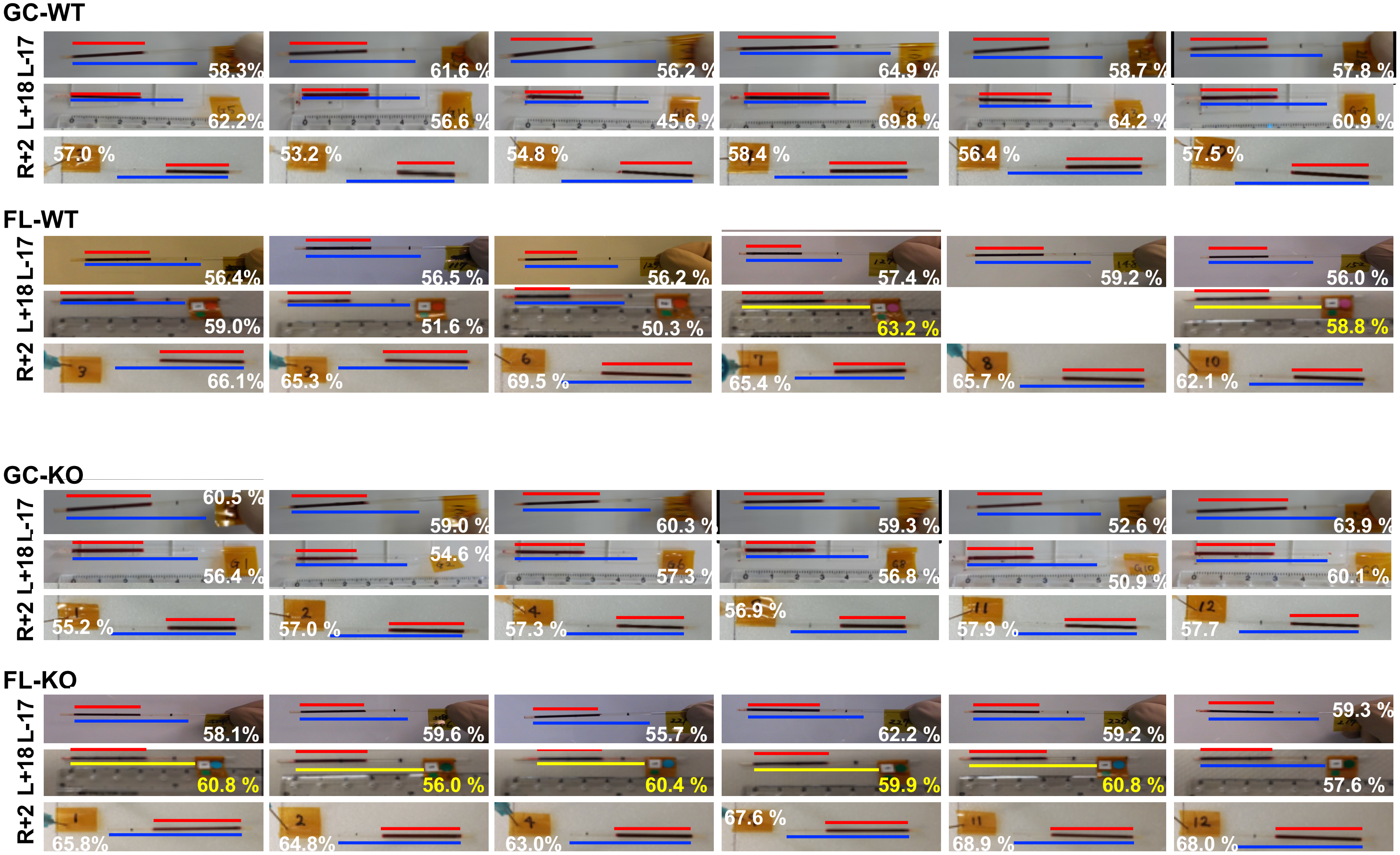

Supplementary Fig. 1. Pictures of the centrifuge tubes used to measure t-Hct.

Pictures of the centrifuge tubes for GC-WT, FL-WT, GC-KO and FL-KO mice at L-17, L+18 and R+2. Red and blue lines indicate the lengths of packed RBCs and whole blood, respectively. The calculated t-Hct values are shown. To our regret, in some samples, the edges of the whole blood were hidden by labels. In such cases, the lengths from the bottom of the blood to the label margin of the tube are indicated by yellow lines. Even in such cases, we calculated the %length of the red line to the yellow line and showed the values individually in yellow. Since the lengths of whole blood are expected to be longer than the yellow line in every tube, the t-Hct values of those tubes are obviously lower than the %length values. Note that we could not obtain tail blood from one FL-WT mouse at L+18.

Supplementary Fig. 2.

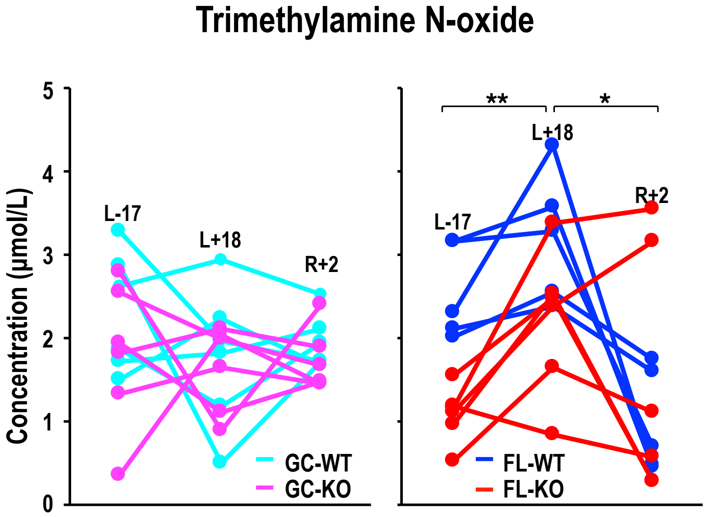

Supplementary Fig. 2. Trimethylamine N-oxide levels are elevated in the plasma during spaceflight.

Time course of changes in the plasma levels of trimethylamine N-oxide at L-17, L+18 and R+2. Data from 6 GC-WT and 6 GC-KO mice are shown in the left panel, and those from 5 FL-WT and 6 FL-KO mice are shown in the right panel. P values obtained with the two-tailed Wilcoxon signed-rank test for the comparison between the levels observed at L-17 and L+18 and those observed at L+18 and R+2 in FL mice are shown. Dots represent individual animals. *, $p < 0.05$; **, $p < 0.01$.

Supplementary Fig. 3.

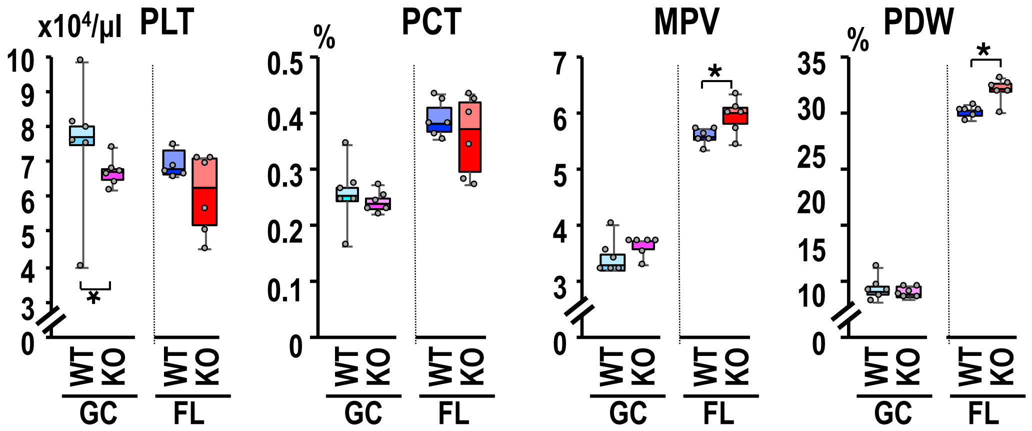

Supplementary Fig. 3. Comparison of platelet parameters between Nrf2-KO and control WT mice in the ground and spaceflight conditions.

Box-and-dot plots of platelet parameters. One-sided Wilcoxon rank sum tests were performed to evaluate the differences between Nrf2-KO and control WT mice in the ground (left part in each panel) and spaceflight (right part) conditions. *; $P < 0.05$.

Supplementary Fig. 4.

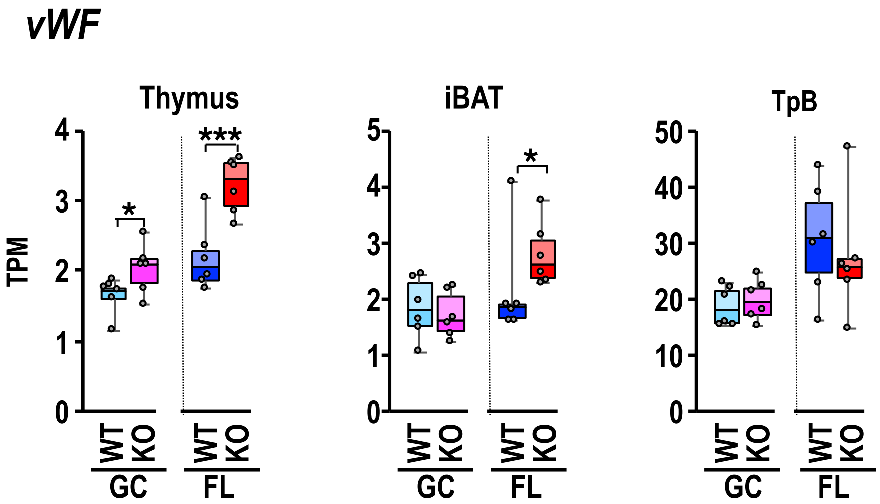

Supplementary Fig. 4. Comparison of *vWF* gene expression between Nrf2-KO and control WT mice in the ground and spaceflight conditions.

Box-and-dot plots of *vWF* gene expression in thymus, interscapular brown adipose tissue (iBAT), and temporal bone (TpB). One-sided Wilcoxon rank sum tests were performed to evaluate the differences between Nrf2-KO and control WT mice in the ground (left part in each panel) and spaceflight (right part) conditions. *; $P < 0.05$, ***; $P < 0.005$.

Supplementary Fig 5.

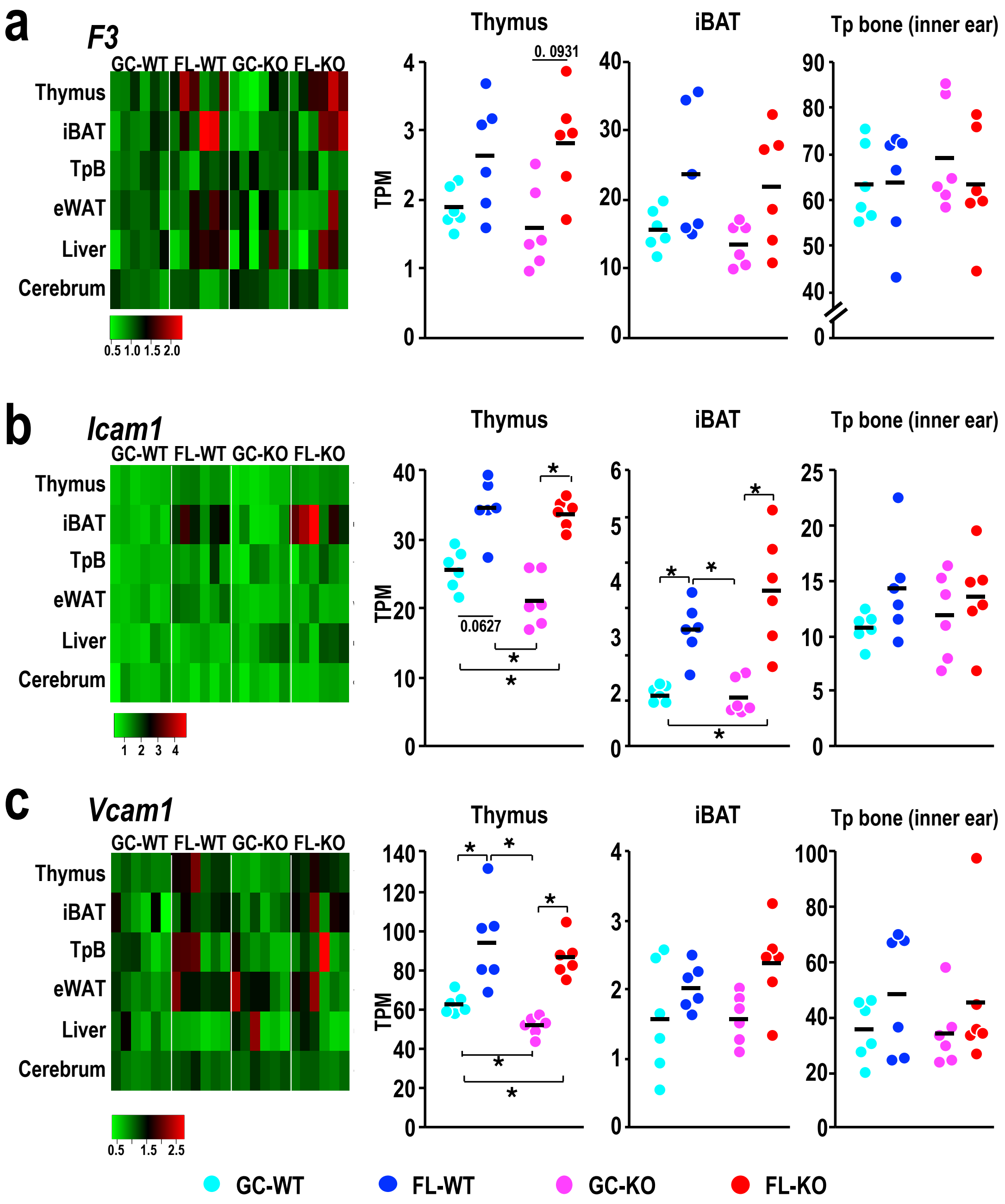

Supplementary Fig. 5. Gene expression changes for tissue inflammation markers.

Expression of the genes encoding F3 (coagulation factor III) (**a**), ICAM1 (**b**), and VCAM1 (**c**) in mice. Heatmaps of the relative expression of these genes in the thymus, iBAT, TpB, epididymal white adipose tissue (eWAT), liver and cerebrum are shown on the left. The mean value of each gene in individual tissues from GC-WT mice was set to one. Dot plots of expression in the thymus, iBat and TpB are shown in the right three panels.

Supplementary Fig. 6.

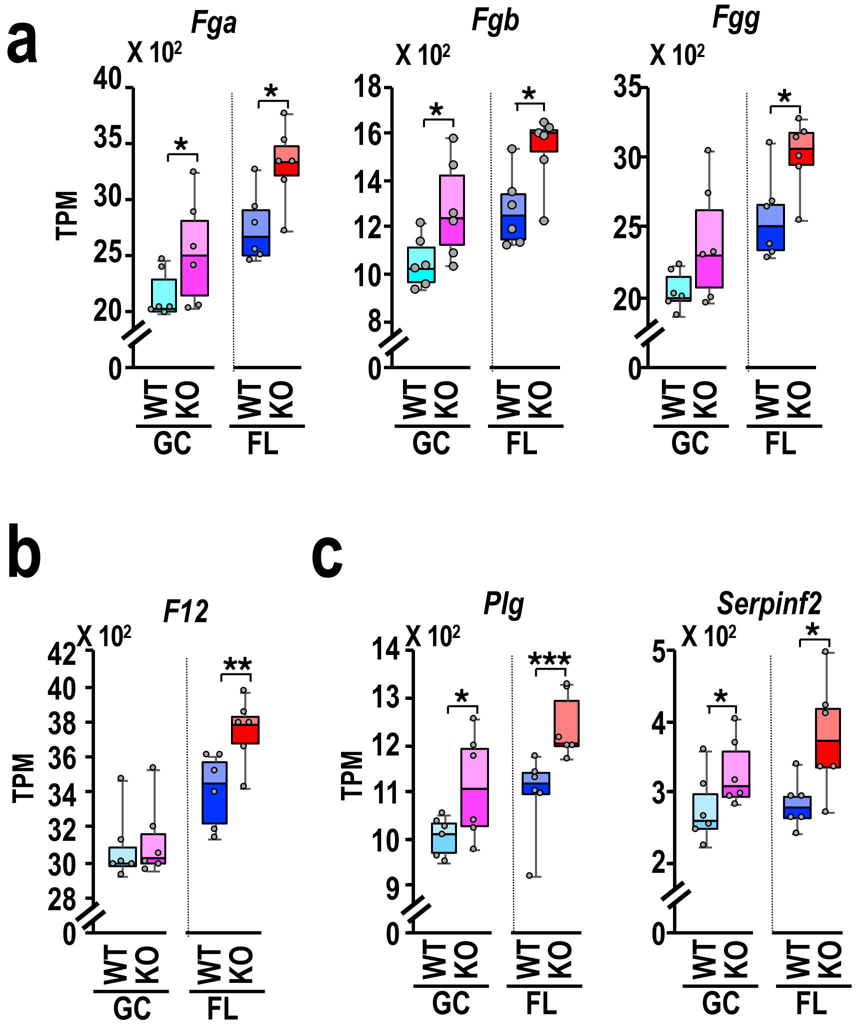

Supplementary Fig. 6. Comparison of coagulation and fibrinolysis gene expression between Nrf2-KO and control WT mice in the ground and spaceflight conditions. Box-and-dot plots of the expression of fibrinogen genes (a), *F12* gene (b) and *Plg* and *Serpinf2* genes (c) in the livers. One-sided Wilcoxon rank sum tests were performed to evaluate the differences between Nrf2-KO and control WT mice in the ground (left part in each panel) and spaceflight (right part) conditions. *; $P < 0.05$, **; $P < 0.01$, ***; $P < 0.005$.

Supplementary Fig. 7.

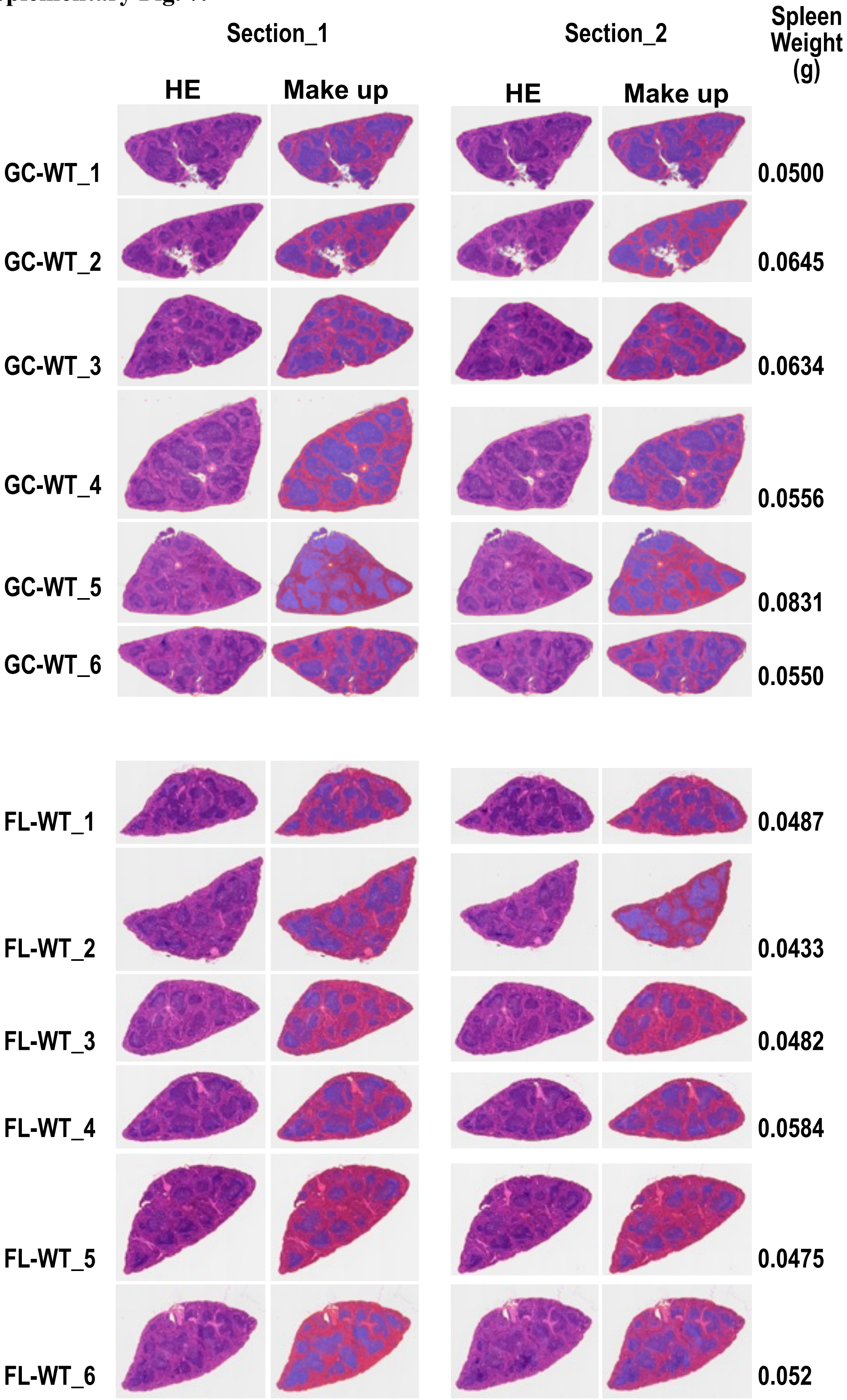

Supplementary Fig. 7. (Continued)

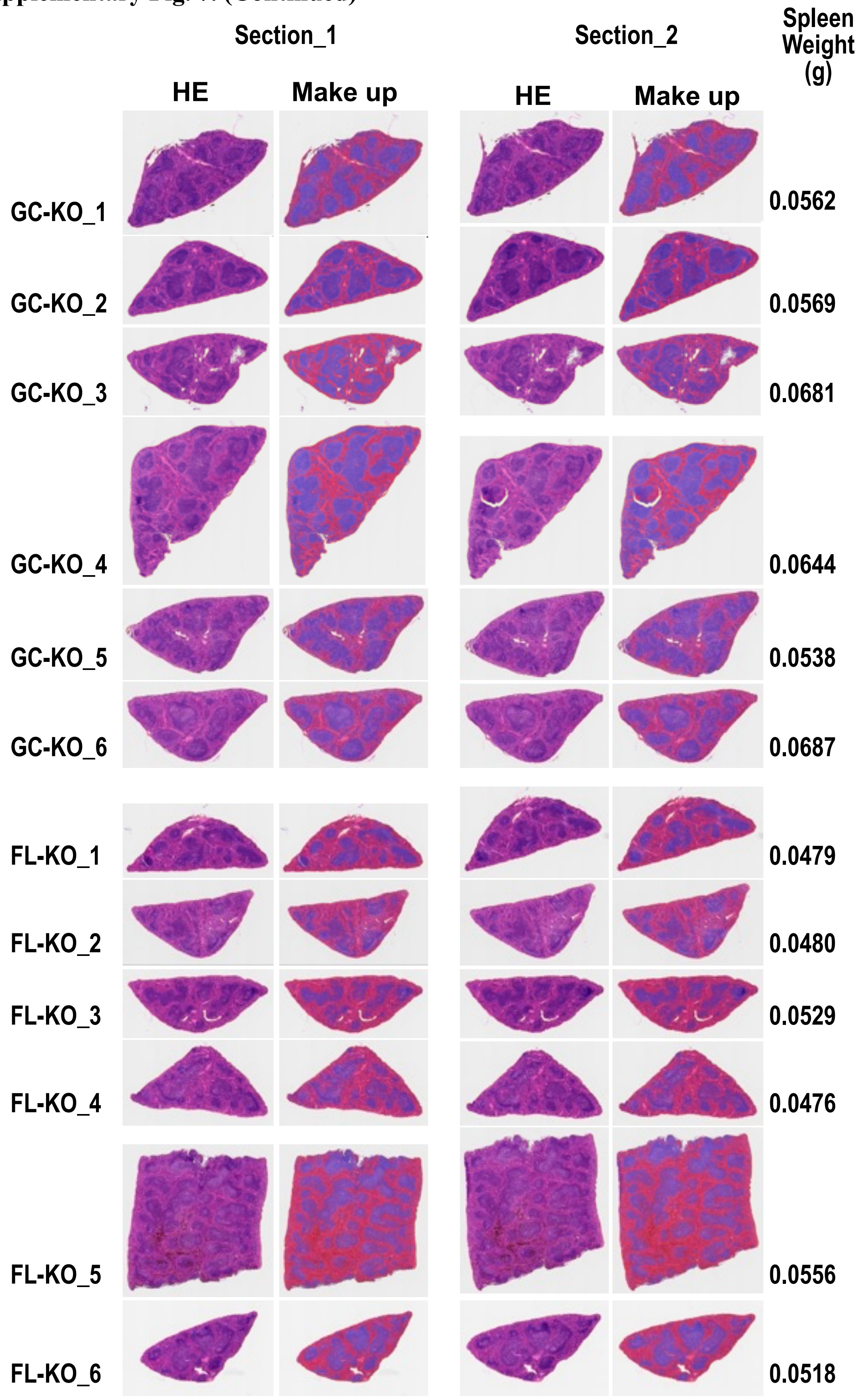

Supplementary Fig. 7. Original and colorized images of HE staining showing WP and RP cell detection and classification.

Original HE staining images (left panels) and colorized images generated using QuPath (right panels) are shown for each of two sections from paraffin-embedded mouse spleens. White pulp (WP) and red pulp (RP) cells are shown in blue and red, respectively, in the colorized images.

Supplementary Fig. 8.

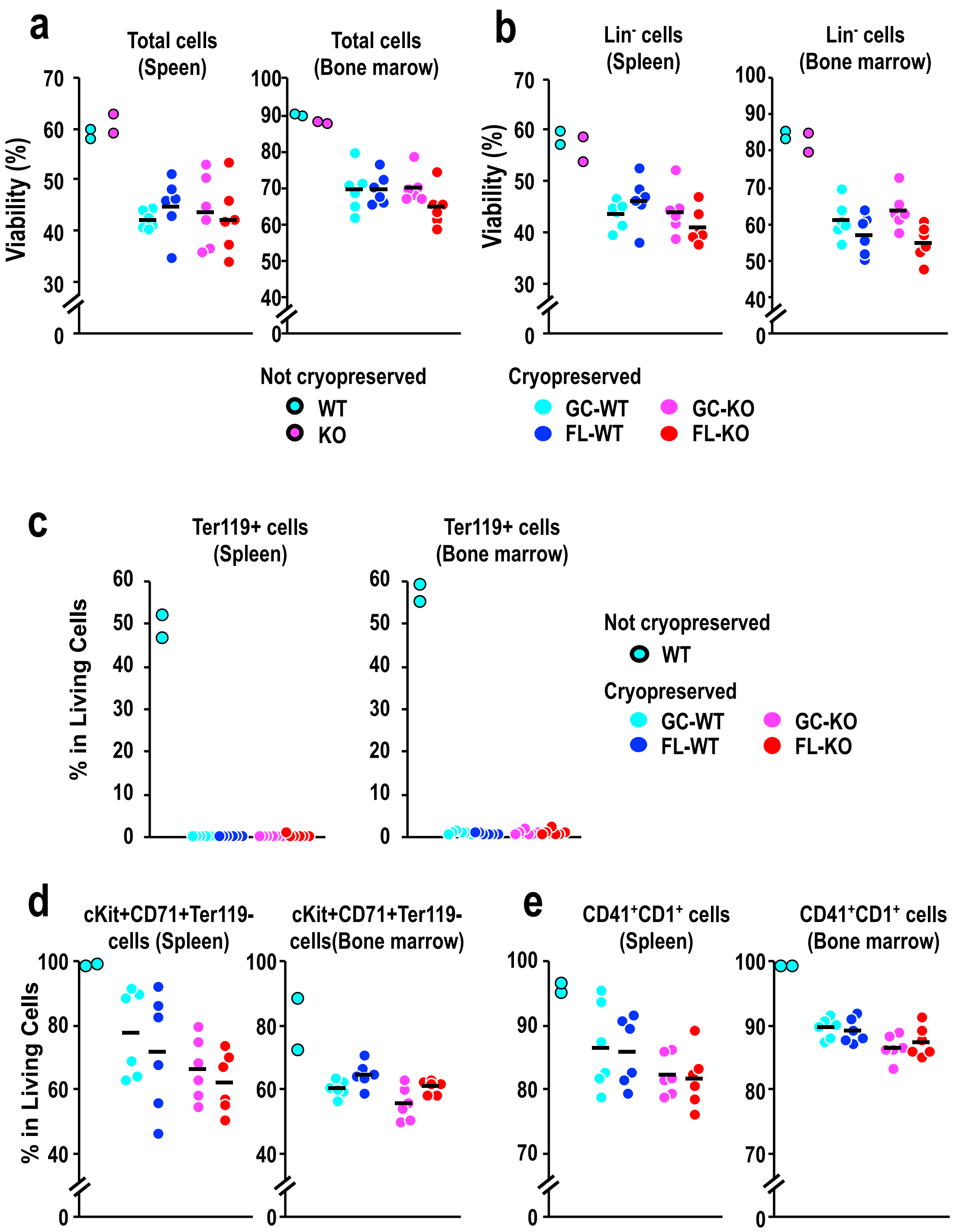

Supplementary Fig. 8. Flow cytometry analyses evaluating cell viability in cryopreserved samples.

Comparison of cell viability for total cells (**a**) and lineage-negative cells (**b**) between fresh and cryopreserved samples from the spleen (left) and bone marrow (right). The percentages of lineage-committed cells among live cells are also shown for Ter119-positive cell populations (**c**), ckit^{+}CD71^{+}Ter119^{-} erythroblast populations (**d**), and CD61^{+}CD41^{+} megakaryocyte populations (**e**). Note that there is a very small population of Ter119^{+} cells among live cells. Dots represent individual animals. Means are shown in the plots.

Supplementary Fig. 9.

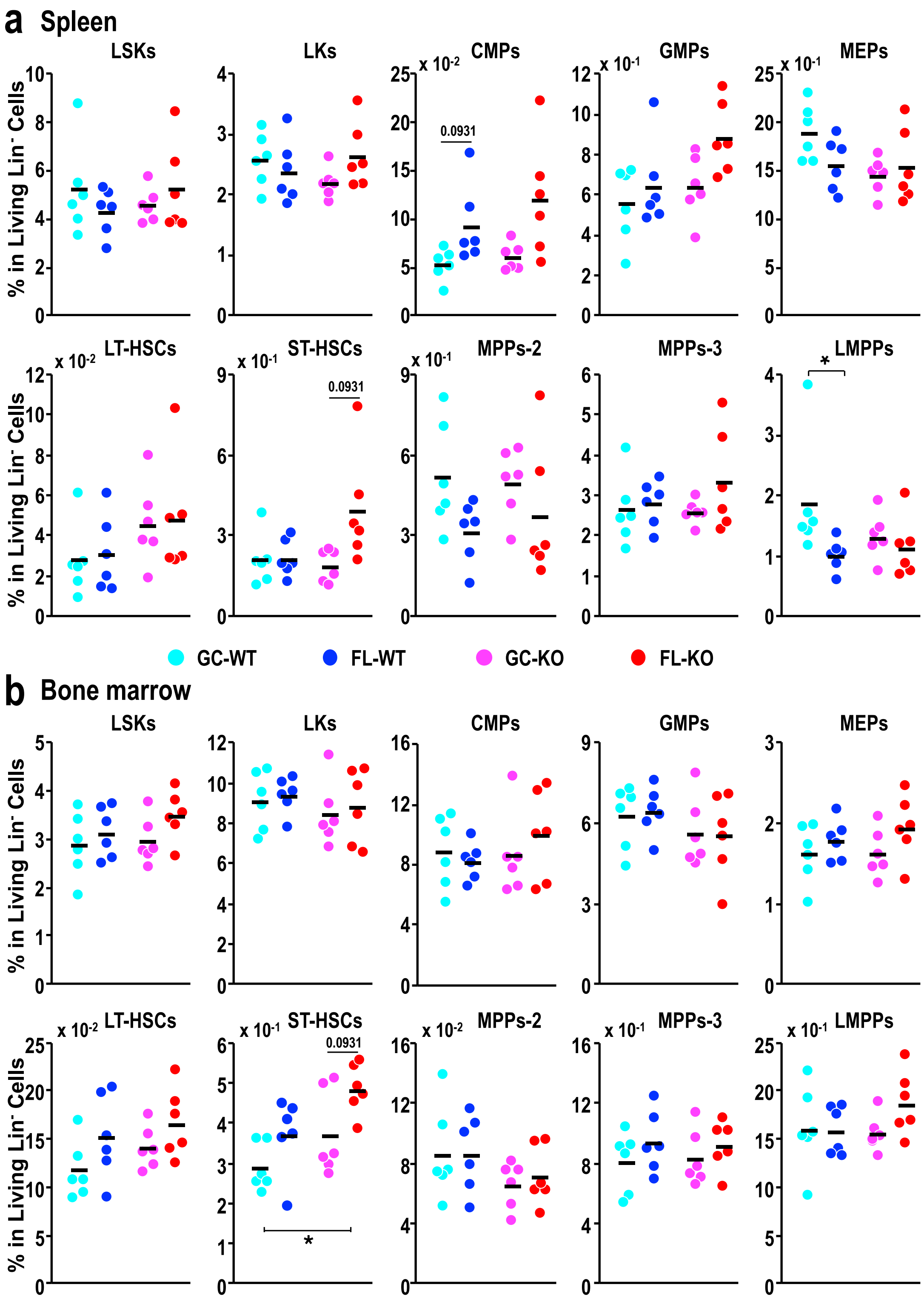

Supplementary Fig. 9. Populations of haematopoietic stem and progenitor cells in the spleen and bone marrow of mice after spaceflight.

Frequencies of various cell populations among live lineage-negative cells from the spleen (**a**) and bone marrow (**b**) are shown. Dots represent individual animals. Means are shown in the plots. *, $p < 0.05$. LSKs, lineage-negative/Sca1-positive/cKit-positive cells; LKs, lineage-negative/Sca1-negative/cKit-positive cells; CMPs, common myeloid progenitors; GMPs, granulocyte-macrophage progenitors; MEPs, megakaryocyte/erythrocyte progenitors; LT-HSCs, long-term haematopoietic stem cells; ST-HSC, short-term HSCs; MPPs-2, multipotent progenitors subset 2; MPPs-3, multipotent progenitors subset 3; and LMPPs, lymphoid-primed multipotent progenitors.

Supplementary Fig. 10.

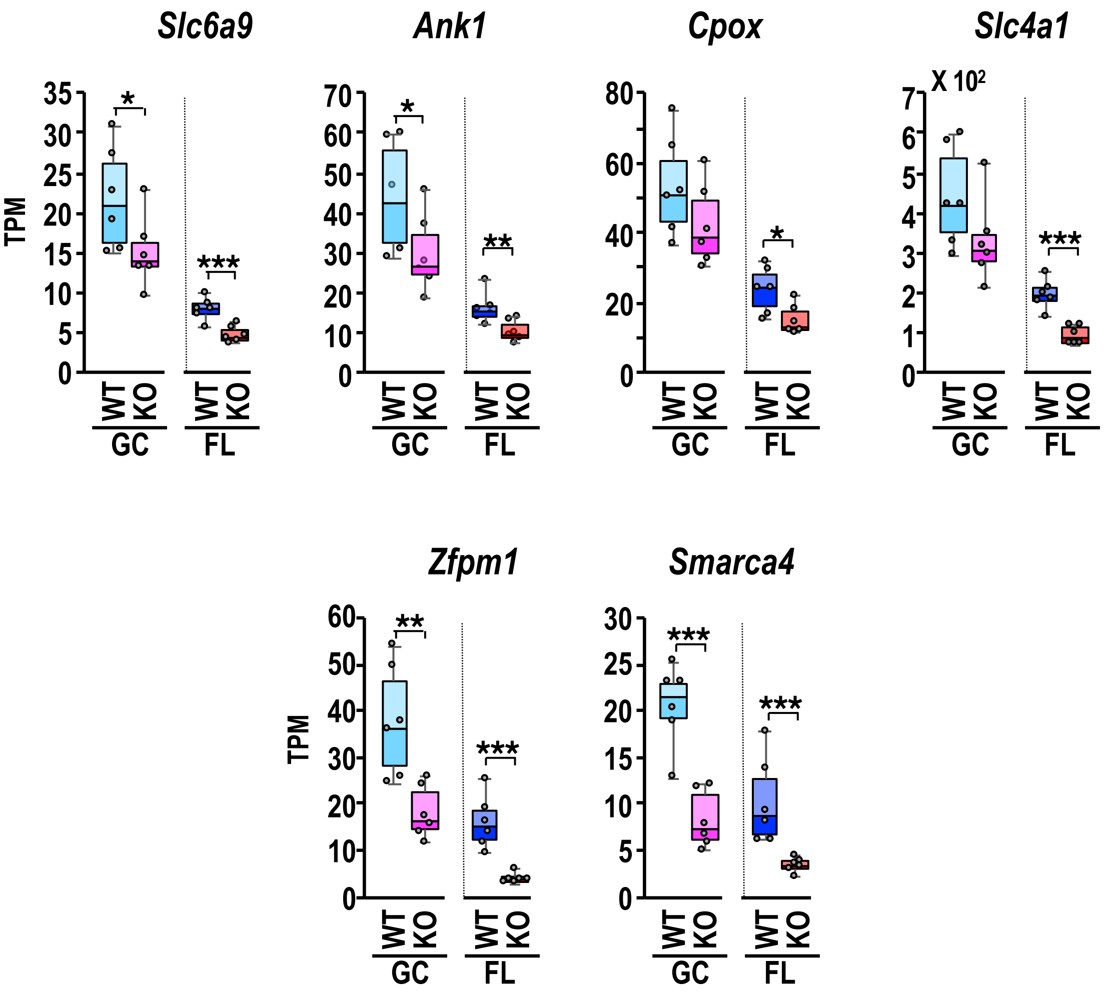

Supplementary Fig. 10. Comparison of erythroid-related gene expression between Nrf2-KO and control WT mice in the ground and spaceflight conditions.

Box-and-dot plots of the expression of *Slc6a9*, *Ank1*, *Cpox, Slc41a*, *Zfpm1* and *Smarca4* genes in the bone marrows. One-sided Wilcoxon rank sum tests were performed to evaluate the differences between Nrf2-KO and control WT mice in the ground (left part in each panel) and spaceflight (right part) conditions. *; P < 0.05, **; P < 0.01, ***; P < 0.005.

Supplementary Fig. 11.

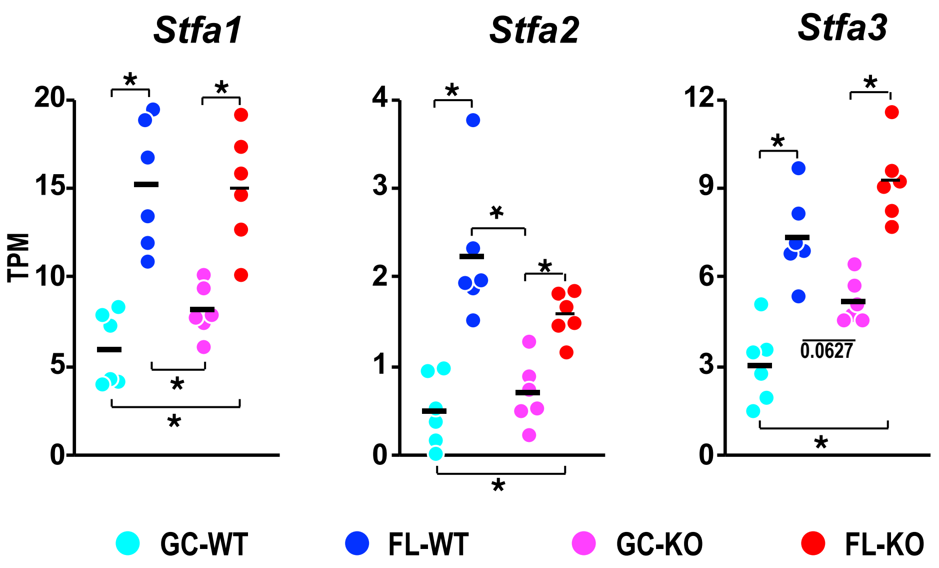

Supplementary Fig. 11. Expression of *Stfa* gene variants in the bone marrow.

Dot plot of the expression of the *Stfa1*, *Stfa2* and *stfa3* genes in the bone marrow. Note that the expression of the three *Stfa* genes was increased by spaceflight. Dots represent individual animals. Means are shown in the plots. *, $p < 0.05$.
