# Supplementary material for: Nrf2 alleviates spaceflight-induced immunosuppression and thrombotic microangiopathy in mice
Source: Commun Biol. 2023 Aug 25;6:875. doi: 10.1038/s42003-023-05251-w (PMC10457343; doi:10.1038/s42003-023-05251-w)
Supplement: Supplementary file 2 — Description of Additional Supplementary Files [file 42003_2023_5251_MOESM2_ESM.pdf]

Description of Additional Supplementary Files

File name: Supplementary Data 1
Description: The source data corresponding to the graphs presented in the main figures and supplemental figures.
