# Supplementary material for: Nrf2 alleviates spaceflight-induced immunosuppression and thrombotic microangiopathy in mice
Source: Commun Biol. 2023 Aug 25;6:875. doi: 10.1038/s42003-023-05251-w (PMC10457343; doi:10.1038/s42003-023-05251-w)
Supplement: Supplementary file 4 — Reporting Summary [file 42003_2023_5251_MOESM4_ESM.pdf]

nature portfolio

Corresponding author(s): Masayuki Yamamoto & Ritsuko Shimizu

Last updated by author(s): Aug 1, 2023

Reporting Summary

Nature Portfolio wishes to improve the reproducibility of the work that we publish. This form provides structure for consistency and transparency in reporting. For further information on Nature Portfolio policies, see our Editorial Policies and the Editorial Policy Checklist.

Statistics

For all statistical analyses, confirm that the following items are present in the figure legend, table legend, main text, or Methods section.

n/a	Confirmed	
☐	☒	The exact sample size (*n*) for each experimental group/condition, given as a discrete number and unit of measurement
☐	☒	A statement on whether measurements were taken from distinct samples or whether the same sample was measured repeatedly
☐	☒	The statistical test(s) used AND whether they are one- or two-sided<br>*Only common tests should be described solely by name; describe more complex techniques in the Methods section.*
☒	☐	A description of all covariates tested
☒	☐	A description of any assumptions or corrections, such as tests of normality and adjustment for multiple comparisons
☒	☐	A full description of the statistical parameters including central tendency (e.g. means) or other basic estimates (e.g. regression coefficient) AND variation (e.g. standard deviation) or associated estimates of uncertainty (e.g. confidence intervals)
☒	☐	For null hypothesis testing, the test statistic (e.g. *F*, *t*, *r*) with confidence intervals, effect sizes, degrees of freedom and *P* value noted<br>*Give P values as exact values whenever suitable.*
☒	☐	For Bayesian analysis, information on the choice of priors and Markov chain Monte Carlo settings
☒	☐	For hierarchical and complex designs, identification of the appropriate level for tests and full reporting of outcomes
☒	☐	Estimates of effect sizes (e.g. Cohen's *d*, Pearson's *r*), indicating how they were calculated

Our web collection on statistics for biologists contains articles on many of the points above.

Software and code

Policy information about availability of computer code

Data collection	The protocols have been described in the "Methods" section, including BD FACSAria II, BD FACSDiva software (Becton Dickinson). Standard, open-source tools used in data collection are described in the "Methods" section.
Data analysis	All computational tools used in data analysis are described in the "Methods" section and are open-sourse tools that have been previously published, including the iDEP.95. Statistical analyses were performed using using JMP Pro 16 (SAS Institute Inc.).

For manuscripts utilizing custom algorithms or software that are central to the research but not yet described in published literature, software must be made available to editors and reviewers. We strongly encourage code deposition in a community repository (e.g. GitHub). See the Nature Portfolio guidelines for submitting code & software for further information.

Data

Policy information about availability of data

All manuscripts must include a data availability statement. This statement should provide the following information, where applicable:

- Accession codes, unique identifiers, or web links for publicly available datasets
- A description of any restrictions on data availability
- For clinical datasets or third party data, please ensure that the statement adheres to our policy

All data reported in this manuscript will be freely available.
The datasets are available form the corresponding author on reasonable request.

March 2021

Human research participants

Policy information about studies involving human research participants and Sex and Gender in Research.

Reporting on sex and gender	n/a
Population characteristics	n/a
Recruitment	n/a
Ethics oversight	n/a

Note that full information on the approval of the study protocol must also be provided in the manuscript.

Field-specific reporting

Please select the one below that is the best fit for your research. If you are not sure, read the appropriate sections before making your selection.

☒ Life sciences ☐ Behavioural & social sciences ☐ Ecological, evolutionary & environmental sciences

For a reference copy of the document with all sections, see nature.com/documents/nr-reporting-summary-flat.pdf

Life sciences study design

All studies must disclose on these points even when the disclosure is negative.

Sample size	Sample-size was determined based on the similar study in the field, and it is indicated either in the figure legends or the methods section.
Data exclusions	No data was excluded for analysis.
Replication	The replication numbers were represented in the figure.
Randomization	Animals were randomly assigned for the different treatment in this study.
Blinding	The investigators were not blinded to group allocation during data collection and analysis.

Reporting for specific materials, systems and methods

We require information from authors about some types of materials, experimental systems and methods used in many studies. Here, indicate whether each material, system or method listed is relevant to your study. If you are not sure if a list item applies to your research, read the appropriate section before selecting a response.

Materials & experimental systems

n/a	Involved in the study	
☐	☒	Antibodies
☒	☐	Eukaryotic cell lines
☒	☐	Palaeontology and archaeology
☐	☒	Animals and other organisms
☒	☐	Clinical data
☒	☐	Dual use research of concern

Methods

n/a	Involved in the study	
☒	☐	ChIP-seq
☐	☒	Flow cytometry
☒	☐	MRI-based neuroimaging

Antibodies

Antibodies used	The detailed information of the antibodies has been introduced in supplementary information.
Validation	All antibodies employed in this study are sourced from the specified commercial providers, as indicated in the supplementary information.

Animals and other research organisms

Policy information about studies involving animals; ARRIVE guidelines recommended for reporting animal research, and Sex and Gender in Research

Laboratory animals	Twelve wild-type and Twelve Nrf2-KO mice in C57BL/6 background were used in this study. The mice were bred at Charles River Laboratories Japan. Six wild-type and six Nrf2-KO mice, aged 8 weeks, were transported to the Kennedy Space Center 3weeks prior to launch. After acclimation in individual housing cages. they were utilized for the space experiment. Additionally, six wild-type and six Nrf2-KO mice were subjected to a ground control experiment at JAXA Tsukuba in Japan, accurately mimicking the conditions of the space experiment.
Wild animals	This study did not involve wild-animals.
Reporting on sex	All mice were male.
Field-collected samples	This study did not utilize samples collected from the field.
Ethics oversight	All animal experiments were approved by the Institutional Animal Care and Use Committees of JAXA (protocol numbers 017-001 and 017-014), NASA (protocol number FLT- 17-112), Explora BioLabs (EB15-010C), and Tohoku University (2017MdA-328) and conducted according to the related guidelines and applicable laws of Japan and the United States of America.

Note that full information on the approval of the study protocol must also be provided in the manuscript.

Flow Cytometry

Plots

Confirm that:

☐ The axis labels state the marker and fluorochrome used (e.g. CD4-FITC).

☐ The axis scales are clearly visible. Include numbers along axes only for bottom left plot of group (a 'group' is an analysis of identical markers).

☐ All plots are contour plots with outliers or pseudocolor plots.

☒ A numerical value for number of cells or percentage (with statistics) is provided.

Methodology

Sample preparation	The detailed information of flow cytometry analyses have been introduced in this study
Instrument	BD FACSAria II
Software	FACSDiva software
Cell population abundance	Details are in the methods section.
Gating strategy	See methods for details.

☒ Tick this box to confirm that a figure exemplifying the gating strategy is provided in the Supplementary Information.
